# Super Resolution Fluorescence Microscopy and Tracking of Bacterial Flotillin (Reggie) Paralogs Provide Evidence for Defined-Sized Protein Microdomains within the Bacterial Membrane but Absence of Clusters Containing Detergent-Resistant Proteins

**DOI:** 10.1371/journal.pgen.1006116

**Published:** 2016-06-30

**Authors:** Felix Dempwolff, Felix K. Schmidt, Ana B. Hervás, Alex Stroh, Thomas C. Rösch, Cornelius N. Riese, Simon Dersch, Thomas Heimerl, Daniella Lucena, Nikola Hülsbusch, Claudia A. O. Stuermer, Norio Takeshita, Reinhard Fischer, Bruno Eckhardt, Peter L. Graumann

**Affiliations:** 1 SYNMIKRO, LOEWE Center for Synthetic Microbiology, Philipps Universität Marburg, Marburg, Germany; 2 Department of Chemistry, Philipps Universität Marburg, Marburg, Germany; 3 Department of Physics, Philipps Universität Marburg, Marburg, Germany; 4 Department of Biology, Philipps Universität Marburg, Marburg, Germany; 5 Department of Biology, University of Konstanz, Konstanz, Germany; 6 University of Tsukuba, Faculty of Life and Environmental Sciences, Tsukuba, Ibaraki, Japan; 7 Department of Microbiology, Institute for Applied Bioscience, Karlsruhe Institute of Technology (KIT), Karlsruhe, Germany; Universidad de Sevilla, SPAIN

## Abstract

Biological membranes have been proposed to contain microdomains of a specific lipid composition, in which distinct groups of proteins are clustered. Flotillin-like proteins are conserved between pro—and eukaryotes, play an important function in several eukaryotic and bacterial cells, and define in vertebrates a type of so-called detergent-resistant microdomains. Using STED microscopy, we show that two bacterial flotillins, FloA and FloT, form defined assemblies with an average diameter of 85 to 110 nm in the model bacterium *Bacillus subtilis*. Interestingly, flotillin microdomains are of similar size in eukaryotic cells. The soluble domains of FloA form higher order oligomers of up to several hundred kDa *in vitro*, showing that like eukaryotic flotillins, bacterial assemblies are based in part on their ability to self-oligomerize. However, *B*. *subtilis* paralogs show significantly different diffusion rates, and consequently do not colocalize into a common microdomain. Dual colour time lapse experiments of flotillins together with other detergent-resistant proteins in bacteria show that proteins colocalize for no longer than a few hundred milliseconds, and do not move together. Our data reveal that the bacterial membrane contains defined-sized protein domains rather than functional microdomains dependent on flotillins. Based on their distinct dynamics, FloA and FloT confer spatially distinguishable activities, but do not serve as molecular scaffolds.

## Author Summary

Many membrane proteins are not uniformly distributed within biological membranes, and may prefer specific lipid environments to function optimally. Using super resolution fluorescence microscopy, we show that several *Bacillus subtilis* membrane proteins indeed cluster into structures of 60 to 110 nm, verifying the existence of defined-size protein microdomains. Biochemical co-isolation of specific membrane proteins and flotillins, a family of proteins highly conserved between eukaryotic and bacterial cells, suggested that common “functional” microdomains exist, containing so-called “detergent-resistant” membrane proteins, that are centered by flotillins. Through high speed tracking of *Bacillus subtilis* FloA and FloT we show that both proteins are not present in the same microdomain, but move through the membrane with different velocities. Dual colour time lapse microscopy showed that contrarily to vertebrate flotillins, bacterial flotillins do not move together with detergent-resistant proteins, ruling out the existence of coclusters. The lack of both flotillins, but not of a single one, leads to striking defects in cell shape and in cell growth, indicating important overlapping functions of flotillin paralogs. Our data show that FloA and FloT perform spatially distinct functions, possibly in the insertion of membrane proteins that require a specific lipid environment, based on a close connection between FloA and FloT with the Sec membrane insertion machinery, but do not act as scaffolds for detergent resistant proteins. Our tracking analyses provide an important basis for the understanding of interactions between membrane proteins in living cells.

## Introduction

In spite of many decades of research on membrane proteins, the true arrangement of proteins and their dynamics within the lipid bilayer are still poorly defined. Many membrane proteins show non-uniform localization patterns [1, 2], and the existence of microdomains having different lipid compositions can be inferred from several lines of experiments [3]. So-called detergent resistant microdomains (DRMs) or lipid rafts have been studied biochemically and cytologically, because they contain a characteristic set of proteins that are involved in a variety of processes [4–8]. However, how lipid domains are set up and are maintained, and how fast they move within the cell membrane remains unclear.

Flotillin/reggie proteins (reggies/flotillins, prohibitins, podocins, stomatins) are an evolutionarily conserved class of proteins found across all organisms [9]. They are considered as regulators of membrane protein trafficking [10] and as common constituents of DRMs in eukaryotic cells. The hallmark of flotillin-like proteins is the SPFH domain (stomatin, prohibitin, flotillin homology) of unknown function, and in general, a single membrane span (with the N-terminus of the protein being on the outside of the cell) in bacterial cells, or no membrane helix but a palmitoyl and myristoyl anchor in eukaryotic cells [11]. In addition to the SPFH domain, flotillin subfamilies contain the so-called flotillin (tail) domain, which is characterized by extended coiled coil motifs and is involved in multimerization [2], but has no known enzymatic function. In eukaryotic cells, flotillins are involved in membrane-trafficking, in signal transduction, and cytoskeletal rearrangement [3, 10]. They are also discussed as scaffolding proteins and as couplers of membrane-proteins with the actin cytoskeleton [12, 13]. During axon growth in neuronal cells, flotillins are suggested to induce membrane microdomain formation at the growth cone [14, 15] and to recruit specific proteins to the elongating axon [16]. Furthermore, flotillins appear to be involved in Alzheimer’s and Parkinson’s disease, and other phenomena [7, 17]. Defects in flotillin proteins are particularly evident in neurons which fail to extend axons and at the recycling compartment of HeLa and A431 cells which fail to properly recycle the transferrin receptor and E-cadherin [10, 18].

In fungi, flotillin proteins are not conserved in budding and fission yeast but are present in ascomycetous filamentous fungi. In the model filamentous fungus *Aspergillus nidulans*, deletion of the flotillin gene, *floA*, impaired a special membrane domain at hyphal tips, which resulted in defects of polarized growth of hyphae, suggesting a conserved function of flotillins in the fungus and in neurons [19, 20].

Bacterial flotillins have so far been characterized in *Escherichia coli*, where a genetic link to a membrane-associated protease was found [21], and in *Bacillus subtilis*, which has two paralogs, FloT and FloA. The absence of one of the flotillins has only minor effects (e.g. a delay in the differentiation process of sporulation [22]), but the absence of both proteins has very severe effects: loss of proper cell shape, a defect in cell division [23], altered membrane fluidity [24, 25], and a defect in signaling events during the transition from planktonic to biofilm growth style [26]. The severity of the defects (including reduced growth rate) highlights the important function of flotillins in bacterial cells.

Using special detergents, *B*. *subtilis* flotillins can be co-isolated with NfeD proteins of unknown function, with the signaling receptor KinC [26], cell wall synthesis enzyme Pbp5, secretory protein SecY, membrane transporters like FhuD, as well as energy metabolism protein AtpDG [27]. Therefore, flotillins have been suggested to set up microdomains within the membrane, by recruiting other proteins and possibly specific lipids into the special structures. It has recently been shown that the overproduction of flotillins increases the stability of a protease, FtsH, within the membrane, which in turn affects cell division and other membrane-associated processes [28]. Indeed, the deletion of both, the gene encoding flotillin T protein in *B*. *subtilis* and of *dynA* (coding for *B*. *subtilis* dynamin) results in cell filamentation [29] and a defect in motility [30]. A flotillin double mutant (*floT* and *floA*) in *B*. *subtilis* also shows a cell filamentation defect [23]. Overproduction of flotillin T results in the considerable shortening of cells [25], supporting the idea that flotillins are directly involved in the division process. Moreover, the absence of flotillin or their overproduction has been shown to affect membrane fluidity [24, 27], and flotillin T has been co-isolated with negatively charged phospholipids, e.g. with phosphatidylglycerol and cardiolipin [22]. The latter is known to facilitate membrane bending, so it is well conceivable that flotillin’s association with this lipid such as cardiolipin facilitates membrane bending, or membrane fusion, possibly performed by dynamin; both processes are crucial steps during bacterial cell division.

Based on conventional fluorescence microscopy, these domains could have a size of 250 nm and more, which are substantial fractions of the surface of a 2 to 4 micrometer large cell. Electron microscopic imaging has shown that flotillin structures are equal or smaller than 100 nm in human cell lines [8, 31]. However, in most cases, flotillins have been visualized by conventional light microscopy, which cannot resolve objects smaller than 250 nm in diameter. Such structures could be interpreted as large raft-like membrane domains. Thus, a description of flotillin assemblies in live organisms is still lacking.

We wished to obtain a better picture of the size and structure of flotillin assemblies, in bacteria as well as in eukaryotic cells, and to study their dynamics in live bacterial cells. We developed a tracking procedure that can accurately determine diffusion constants of proteins moving along a curved membrane, and provide data on the time scale in which flotillin assemblies can meet in the membrane, or in which flotillin structures can cover the entire surface of a cell with regard to their size and diffusion rates. By comparing the movement of flotillins and other proteins proposed to be a part of DRMs, we show that in spite of the formation of conserved-sized individual protein domains, these do not track together, indicating that many bacterial proteins are self-organized into defined assemblies, but that mixed protein compartments only exist transiently.

## Results

### Flotilin A and T localize in the membrane as defined assemblies with an average size of 80 to 90 nm

To gain insight into the nature of bacterial flotillin assemblies, which have been speculated to constitute lipid microdomains, we employed stimulated emission depletion (STED) superresolution fluorescence microscopy. Using G-STED, we have achieved a resolution of 42 nm for MreB filaments in live *B*. *subtilis* cells [32]. We visualized two flotillin-YFP fusions, FloA-YFP and FloT-YFP, in live, exponentially growing or stationary phase *B*. *subtilis* cells. Both fusions were shown to functionally replace the wild type proteins, when expressed from the native gene locus, or from an ectopic site on the chromosome, in previous work [22, 23, 26]. For example, cells carrying deletions of both, *floA* and *floT* genes, grow much slower than wild type cells, are twisted and bent and highly elongated, while single *floT* or *floA* mutant cells do not show any of these strong phenotypes. Likewise, cells lacking *floA* and expressing FloT-YFP (or FloT-mVenus), or vice versa, grow like wild type cells. Thus, the fluorescent protein fusions are able to fulfill the functions of the wild type proteins.

**Fig 1:**
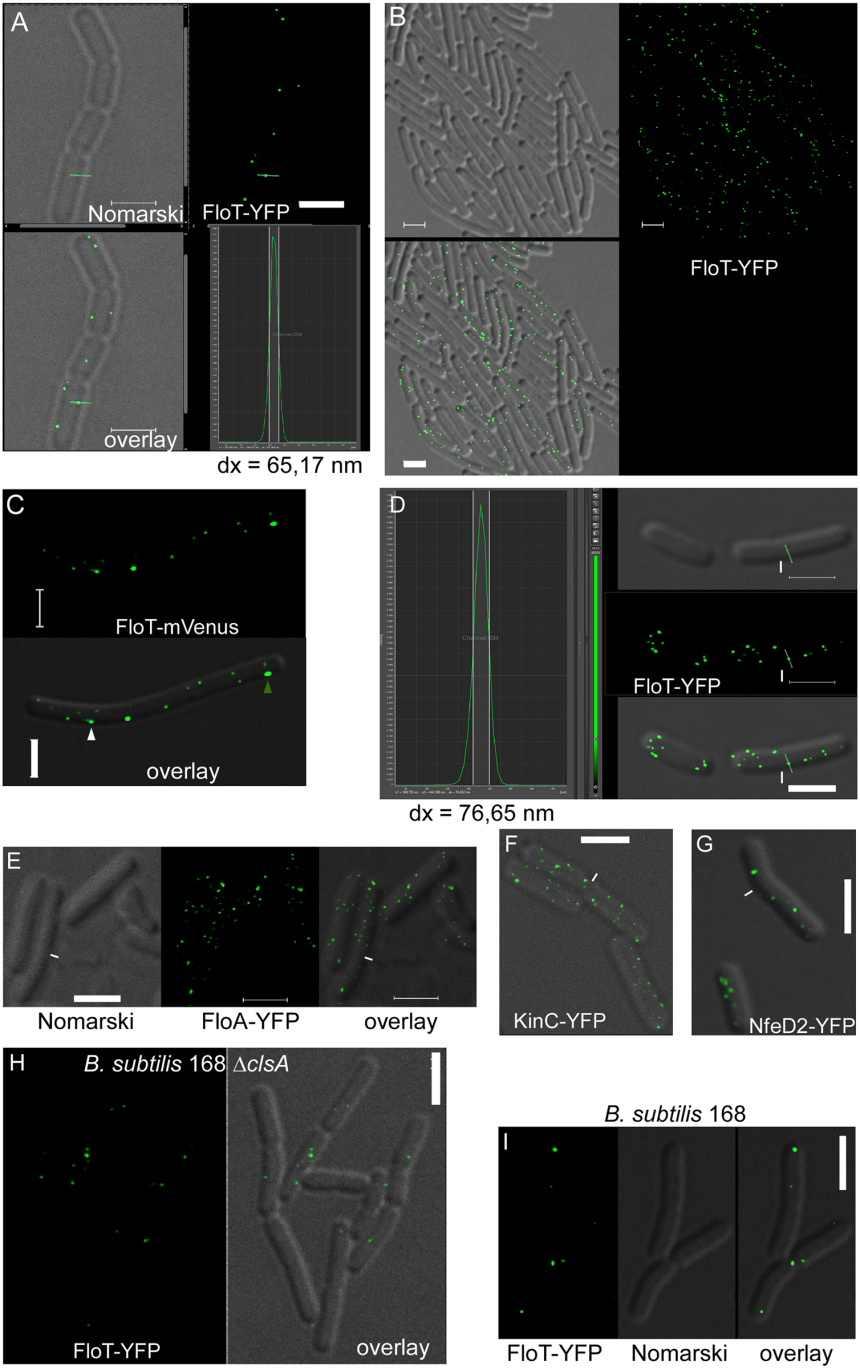
Localization of *Bacillus subtilis* proteins in live cells by STED microscopy. A) localization of FloT-YFP in exponentially growing cells (PY79), lower left panel overlay of Nomarski DIC and fluorescence, green bar indicates focus whose diameter is measured; lower right panel fluorescence intensity plot showing the emission of the measured focus, width of 50% fluorescence intensity (which contains more than 90% of the chromophores) is shown by the white lines, Dx denotes measurement of width in nm. B) STED image of FloT-YFP expressed from the amylase locus in a merodiploid strain. C) localization of FloT-mVenus in exponentially growing cells; white triangle indicates a focus that moves during acquisition, having a characteristic comet tail, green triangle indicates a signal that is ovoid, indicating a non-round assembly. D) localization of FloT-YFP in stationary phase cells, labeling analogous to panel A). E) Localization of FloA-YFP in exponentially growing cells, F) KinC-YFP in exponentially growing cells, G) NfeD2-YFP in exponentially growing cells. H) Strain 168, carrying a deletion in the major cardiolipin synthetase (*clsA*) expressing FloT-YFP, I) Strain 168, expressing FloT-YFP. White lines indicate ends of cells, scale bars 2 μm.

For both fusions, we rarely observed structures of 50 nm in diameter (Fig 1A), but usually larger sized foci, determined using the Leica software (see below), showing that the assemblies are above the resolution of STED microscopy. FloT has been shown to move within the membrane in a time frame of a few seconds, with some static and some motile assemblies [23], potentially causing a drift during acquisition time. Round objects that move during confocal acquisition have a characteristic comet-like structure. We used a scan speed of 400 Hz, which is fast enough to localize flotillin-YFP foci with high efficiency, because we observed few foci with a comet-tail (indicated in Fig 1C); such cases were rare (4%, n = 250 foci analysed). It should be noted that many flotillin foci were oval or irregularly shaped (Fig 1C), suggesting that not all assemblies are uniformly round. Therefore, our experiments adequately reflect the size of flotillin-YFP assemblies in live cells.

Interestingly, a majority of FloT-YFP signals had a size of more than 50 nm. We measured 225 foci from 125 cells and determined an average size for FloT-YFP assemblies of 85.3 nm ± 12.5 nm (SD) (Fig 1A), and a maximal size of 97.0 nm. The size of fluorescent foci was very similar between FloT-YFP expressed from the original gene locus, or expressed ectopically from the amylase locus (Fig 1B). Thus, FloT assemblies have a relatively uniform size in live cells. We also imaged a monomeric variant of YFP, mVenus, fused to FloT (Fig 1C), which yielded a size distribution that closely resembled that of FloT-YFP, ruling out an artifact from FP-induced multimerization. The diameter of FloT-YFP foci did not change considerably between exponential growth and stationary phase (Fig 1D), but the number of foci increased during the transition to stationary growth, as was reported earlier [23]. We performed the same experiments for the second flotillin-like protein in *B*. *subtilis*, FloA. Like the FloT-YFP fusion, a FloA-YFP fusion can functionally replace the original protein [23, 26]. Interestingly, FloA-YFP foci had an average size of 80.4 nm ± 10.9 nm (SD) (Fig 1E), and thus displayed a similar size distribution like FloT-YFP. These experiments reveal that bacterial flotillin-like proteins have a preferred size for their assemblies, and suggest that flotillins form defined assemblies, however with a considerable size-variation.

### The size of FloA and FloT assemblies varies between different *B*. *subtilis* strains but is robust in different lipid mutant backgrounds

Under standard (exponential) growth conditions, bacteria have a preferred composition of the membrane in terms of ratios of different lipids. We wished to gain insight into the question whether flotillin assemblies may be influenced in number and/or size in cells lacking different lipids. We used four strains that have been generated for *B*. *subtilis* by the Helmann group, namely carrying a deletion of the major cardiolipin synthetase (*clsA*) [33], a deletion in the phosphatidyl-ethanolamine pathway (*pssA*), a deletion in the lysylphosphatidyl-glycerol pathway (*mprF*), or having a lack of glycolipids (*ugtP*) [34]. We used *B*. *subtilis* strain W168 as background, rather than PY79. Interestingly, we found that flotillin assemblies are larger in strain 168 than in strain PY79: FloA-YFP foci were 109 ± 9.7 nm (S1A Fig), while FloT-YFP foci were of a size of 106.7 ± 12.7 nm (Fig 1I, n = 38). These results show that strain backgrounds can have an effect on the size of flotillin clusters. Next, we introduced FloA-YFP and FloT-YFP fusions into the different mutant backgrounds and analysed their localization. Glycolipid mutant cells displayed reduced and abnormal cell size/shape (S1B and S1H Fig), all other mutant cells had wild type-like appearance. There was no visible difference in the apparent size of the clusters (S1B to S1H Fig), which was confirmed by STED measurements: flotillin focus sizes varied between 105 and 115 nm (Table 1), revealing that the assembly of flotillin domains is not influenced by the lack of the different lipids tested. However, the experiments do not rule out other, more specific lipid requirements for the regulation of flotillin oligomerization.

**Table 1:** Size of flotillin clusters in *B*. *subtilis* W168 wild type and lipid mutant cells.

strain	Size of assemblies (nm)	SD (nm)	n
*floA-yfp* (wild type)	109.3	9.7	38
*floT-yfp* (wild type)	106.7	12.7	36
*ΔclsA floA-yfp*	114.6	10.1	35
*ΔclsA floT-yfp*	106.2	12.1	35
*ΔmprF floA-yfp*	108.5	11.0	35
*ΔmprF floT-yfp*	105.8	9.8	35
*ΔpssA floA-yfp*	106.2	9.9	35
*ΔpssA floT-yfp*	106.7	10.5	35
*ΔugtP floA-yfp*	115.5	13.0	35
*ΔugtP floT-yfp*	113	10.6	35

### The soluble domains of flotillin A form large multimeric structures *in vitro*

**Fig 2:**
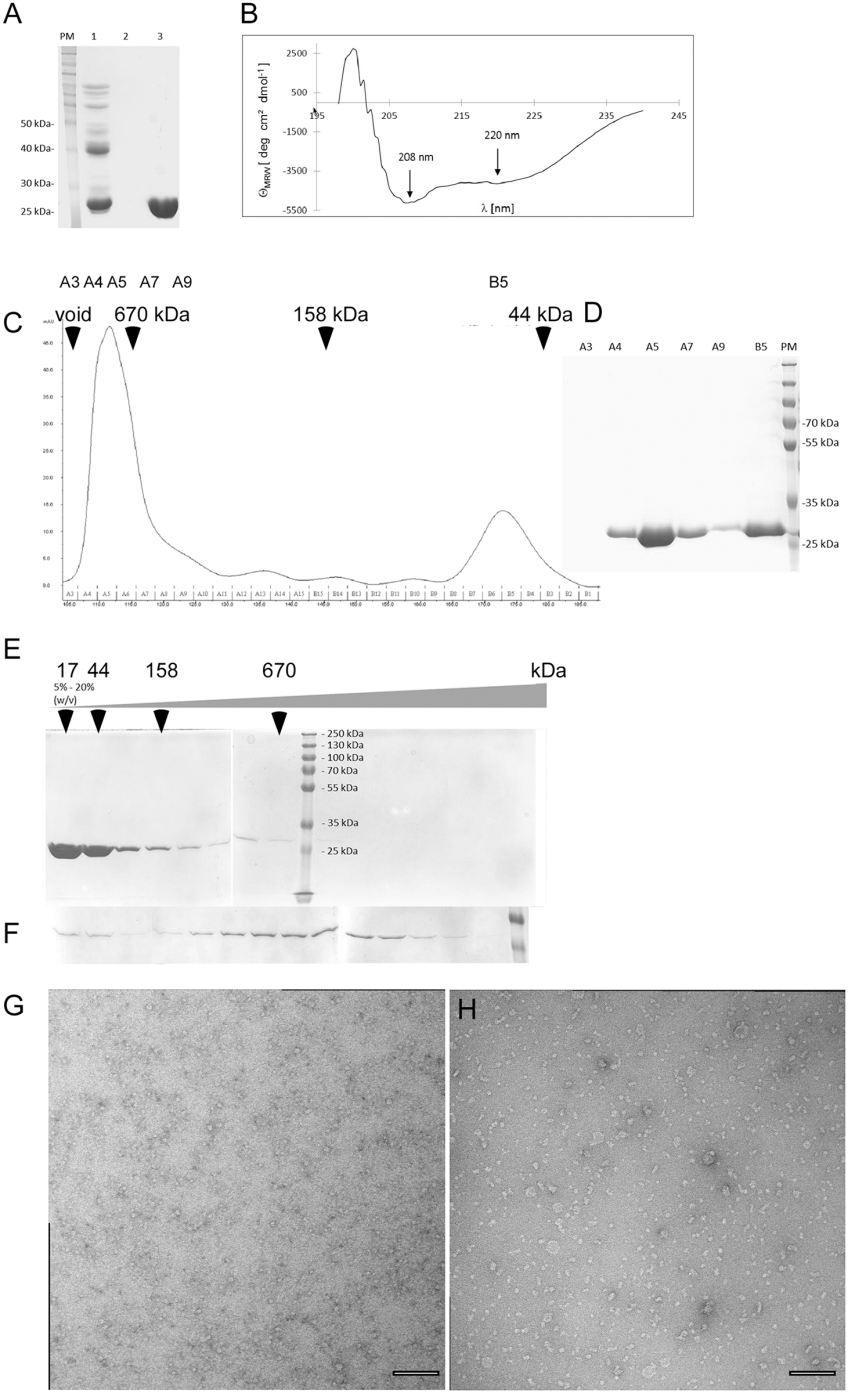
The soluble part of FloA (FloAsp) forms multimers. A) SDS-PAGE showing purification by Ni-NTA chromatography of FloAsp carrying a 6xHis tag at the N-terminus, lane 1 flow through, lane 2 wash step, lane 3 peak elution. B) Circular dichroism analysis of FloAsp (25 μM). C) Size-exclusion chromatography (SEC) of FloAsp; elution of marker proteins and of the void peak are indicated by arrows, numbered letters indicate fractions, which are shown in D) SDS PAGE showing corresponding fractions from SEC. E-F) SDS PAGE of fractions from 5 to 20% (w/v) sucrose gradient centrifugation, position of marker proteins is indicated by arrows, size of denatured proteins is indicated by the marker lane. E) Loading of fraction B5 (dimeric FloAsp) from SEC, F) loading of fraction A5 (multimeric FloAsp); the last lane of the SDS PAGE shows the 35 and 25 kDa proteins from the marker lane. G) Electron microscopy (EM, uranyl acetate negative stain) of the LMW fraction of Strep-FloAsp, H) EM of the HMW fraction of Strep-FloAsp, scale bars 100 nm.

Flotillin/reggie proteins are notoriously difficult to purify for biochemical experiments. We were successful in purifying the soluble part of FloA (Fig 2A) via Ni-NTA chromatography. CD analysis showed that the soluble part has a defined fold, with mostly alpha helical arrangement (60.8%), and few beta-sheet elements (prediction 7.1%, using the GOR IV secondary structure prediction program (https://npsa-prabi.ibcp.fr) (Fig 2B). This is in agreement with the predicted and solved structures of the SPFH domain, which contains a β-sheet, and otherwise a large number of α–helices [35, 36], and with the prediction from coiled-coil analysis software that the flotillin domain contains a high degree of heptad-rich repeats [23]. To investigate if the assembly of flotillins into the defined structures is mediated through self-interaction, we performed analytical gel filtration (GF) and sucrose gradient centrifugation. The protein eluted from gel filtration just behind the void volume, in a peak above 670 kDa, besides a smaller low molecular weight (LMW) fraction (Fig 2C and 2D), whose size corresponds to a FloA dimer (60 kDa). These data suggest that the soluble part of FloA forms predominantly multimeric structures. To rule out artefacts caused by the use of a hexa-histidine affinity tag, we also purified FloA using a strep-tag, which yielded a similar preference for the formation of high molecular weight (HMW) polymeric structures eluting over a wide range of sizes, and a much smaller LMW part eluting around 120 kDa (S2A Fig), which would correspond to a tetramer of FloA as smallest unit. It is possible that the histidine-tag weakens tetramer formation. We used the high or low molecular weight fractions from GF columns for sucrose gradient experiments. Interestingly, when the LMW fraction of FloA was analyzed for its distribution in sucrose gradients, most of the protein sedimented as dimers or tetramers, and some degree of multimerization was observed (Fig 2E) Likewise, when the multimeric fraction was separated on a sucrose gradient, only a small amount of LMW protein arose, and a majority of molecules remained in the multimeric state, with a peak above 670 kDa (Fig 2F). To obtain more insight into the exchange between multimeric assemblies and smallest FloA units, different concentrations of HMW or of LMW fractions of FloA-Strep were subjected to GF analysis. S2B Fig shows that 3 fold and 5 fold dilution of the HMW fraction leads to a concomitant decrease in HMW and in LMW formation. We measured the peak area of both fractions, which showed that in undiluted or 3-fold diluted conditions, about 90% of the proteins are in the HMW fraction, and in the 5-fold diluted condition, 82% are in the HMW fraction and 12% in the LMW peak. A 2 fold concentration of the LMW fraction leads to an increase in formation of multimers relative to the LMW fraction, albeit only of multimers up to 670 kDa (S2C Fig). These experiments show that there is an equilibrium between the smallest unit and multimeric fractions; the tetramers readily form multimers when the concentration is increased, and the equilibrium is far on the multimer side, showing that these are rather stable structures. If only peak fractions from both methods are taken into account for native molecular mass calculation (750 kDa from GF, 750 kDa from sucrose gradients), the formation of 25mer structures can be deduced, but the size variation is very large, such that 12mer up to 50mer assemblies can be observed *in vitro*. The large variation of subunit number was also observed using electron microscopy (EM), after uranyl acetate negative staining of purified FloA multimers. When the LMW fraction was used for EM, homogeneous small structures were observed (Fig 2G), whereas a wide range of different sizes of multimeric particles exists when the HMW fraction was imaged (Fig 2H and S3 Fig), the largest of which had a size of 70 nm.

These experiments show that the flotillin structures observed *in vivo* are in part mediated through flotillin self-interaction, as shown for flotillin and prohibitin in vertebrate cells [2, 37].

### Flotillin protein domains contain dynamically exchanging multimers

**Fig 3:**
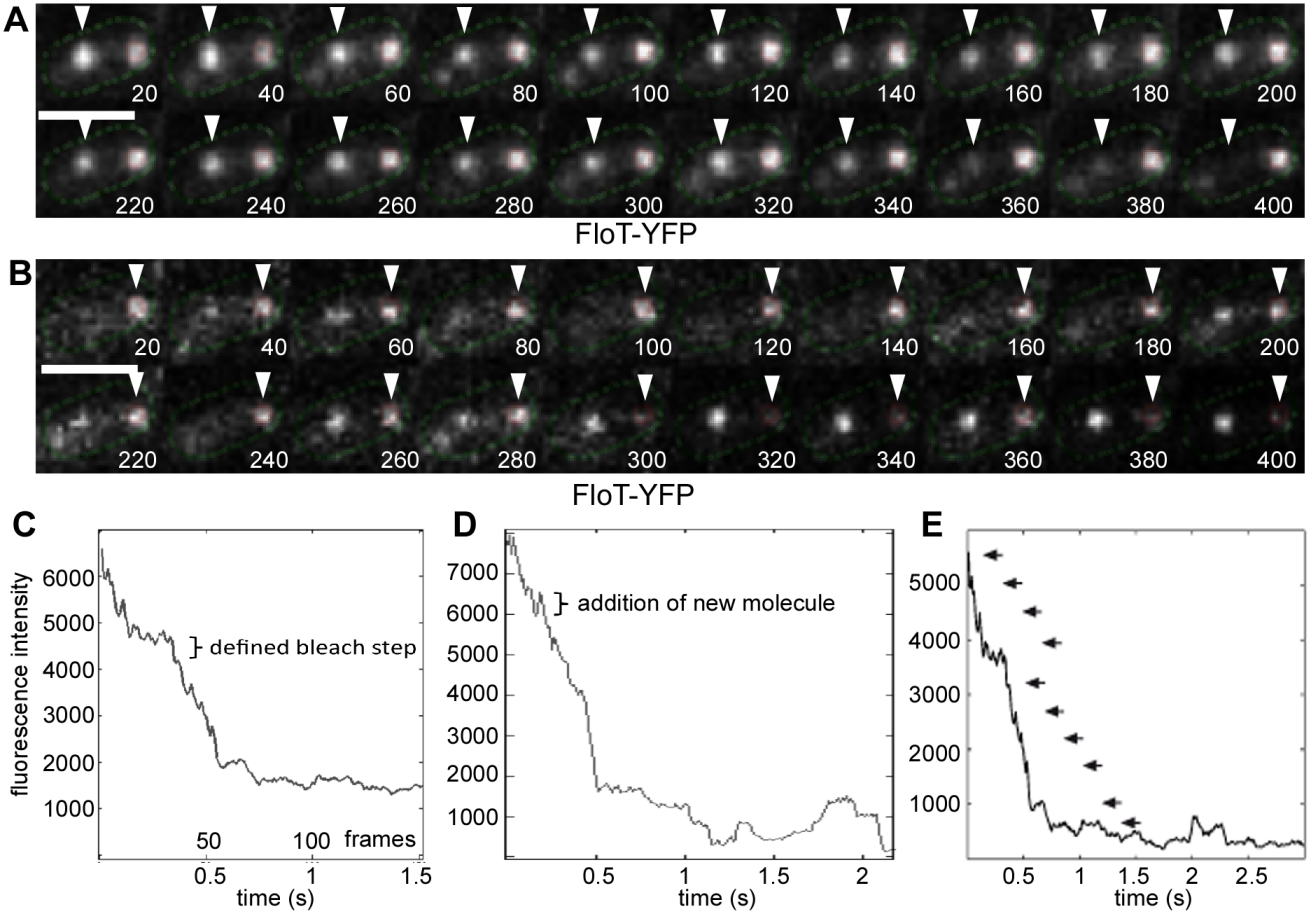
Determination of subunit stoichiometry using single molecule microscopy. A) and B) 20 consecutive images of a streams acquisition of one cell expressing FloT-YFP, 20 ms time intervals. The triangles indicate a relatively stationary focus that bleaches during the stream acquisition. White bar 2 μm. C to E) Graphs showing bleaching events. One individual bleaching step is indicated in panel C), an event of addition of a molecule (positive increase in fluorescence) in panel D), panel E) shows all clear bleaching steps in an acquisition.

To investigate if flotillin foci are indeed composed of a considerable number of monomers, we performed single molecule microscopy, in which cells are exposed to a focused laser illumination. Bleaching of fluorescent spots is monitored in stream acquisitions using a fast EM-CCD camera, such that single bleaching steps can be monitored [38, 39] (S1 Movie). Gradual bleaching of molecules within a fluorescent focus leaves single fluorescent-protein spots that bleach in a single step, revealing the intensity of a single chromophore. Initial intensity of spots can then be used to determine the number of fluorescently-labeled subunits. Fig 3A and 3B show representative images from an experiment, in which initial fluorescence in two foci is reduced to a single spot that finally bleaches in one step. In Fig 3C to 3E, examples for bleaching kinetics of two FloT-YFP spots are shown, in which the intensity of a single YFP molecule can be seen towards the end of the acquisition, and total intensity of the spot in the first frames. FloT-YFP expressed as sole source in the cell showed an average of 12 bleaching steps in 30 spots analysed, revealing that at least 12 molecules are present within the 75 nm structures. The true number of molecules is somewhat higher, because a subset of fluorophores will not have matured, or may have been bleached at an earlier time point in the acquisition (i.e. before the first frame that is used in the analysis). FloA-YFP foci had a fluorescence intensity that was very close to that of FloT-YFP, and therefore, also FloA assemblies will contain a number of monomers, in agreement with the formation of multimers *in vitro*. Interestingly, fluorescent foci frequently showed regain of fluorescence (S1 Movie and Fig 3D and 3E), revealing that non-bleached molecules can be recruited into the assemblies, which occurred in 50% of the analysed spots. Bleaching occurred over 100 to 150 frames, i.e. within 2 to 3 s, showing that flotillin assemblies have subunit exchange within the frame of few seconds.

### Flotillin microdomains in eukaryotic cells are of similar size as their bacterial counterparts

Flotillin microdomains have been visualized and measured by various microscopic methods. The most accurate method used so far, electron microscopy, revealed assemblies of down to 100 nm in human cell lines. Because STED is highly suitable to determine the size of flotillin assemblies in live cells, we analysed FloA-GFP in the model filamentous fungus, *Aspergillus nidulans* [19], and reggie-1 (flotillin-2)-GFP labeled human cells.

**Fig 4:**
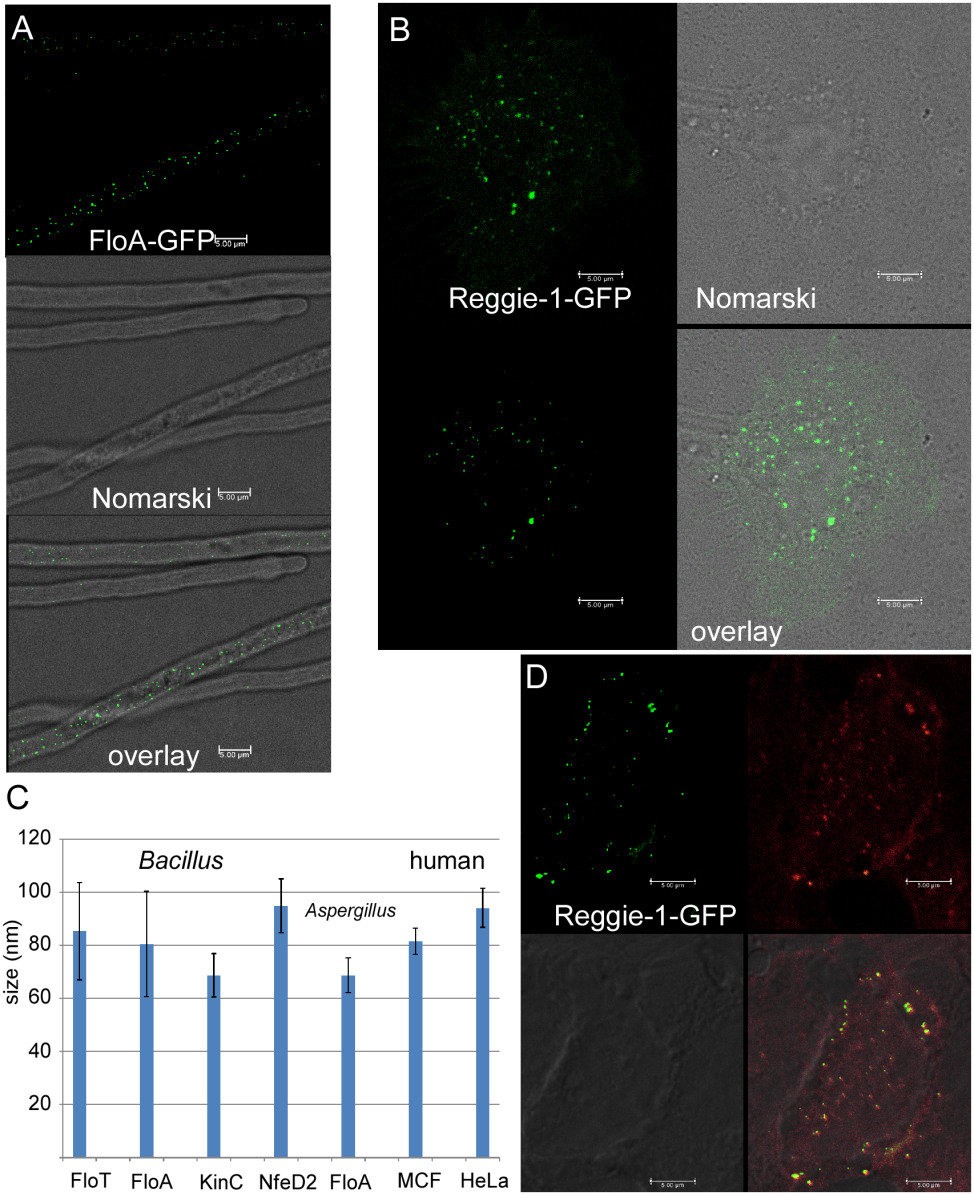
Localization of eukaryotic flotillins, imaged by STED microscopy. A) FloA-GFP expressed in *Aspergillus nidulans*, B) reggie-1 (flotillin-2)-GFP in HeLa cells, C) Sizes of flotillin assemblies in various organisms, standard deviation is indicated by bars, n = 50 or more per protein, D) reggie-1 (flotillin-2)-GFP in MCF-7 cells.

In the hyphae of *A*. *nidulans*, FloA-GFP was detected as defined foci along the hyphal cortex but was excluded from the hyphal tip [19]. In young fungal hyphae, flotillin foci were present as defined foci, with an average diameter of 68.7 nm ± 8.1 nm (SD) (Fig 4A and 4C). Older hyphae were visually distinct from younger ones in that they contained large vacuoles, and contained more flotillin signals (Fig 4A), which could reach sizes of 150 nm. The reason for this increase in size dependent on hyphal age is unknown. Possibly, due to the visual increase in number, closely adjacent assemblies can appear as a large double-sized structure. In any event, in cells having fewer and well-spaced foci, flotillin assemblies have a size that is very similar to that of bacterial flotillin.

In HeLa cells, flotillin is found at the cytoplasmic face of the plasma membrane, but also at various internal membrane structures [10] (Fig 4B). For simplicity, we focused our measurements on flotillin assemblies at the cell membrane, which was ensured through making a Z-stack using conventional confocal images, and a G-STED image at the cell periphery (Fig 4B). Flotillin-2-GFP signals also had an average size of 94.1 nm ±7.4 nm (SD), and were thus also of relatively well defined size. In MCF-7 cells, flotillin signals measured only 81.5 ± 5.0 nm (SD) (Fig 4D and 4C), even more closely resembling bacterial flotillin assemblies (Fig 4C). Therefore, the average size of flotillin structures in eukaryotic cells is quite conserved and comparable to those in bacterial cells.

### Flotillin paralogs move at different velocities through the membrane

**Fig 5:**
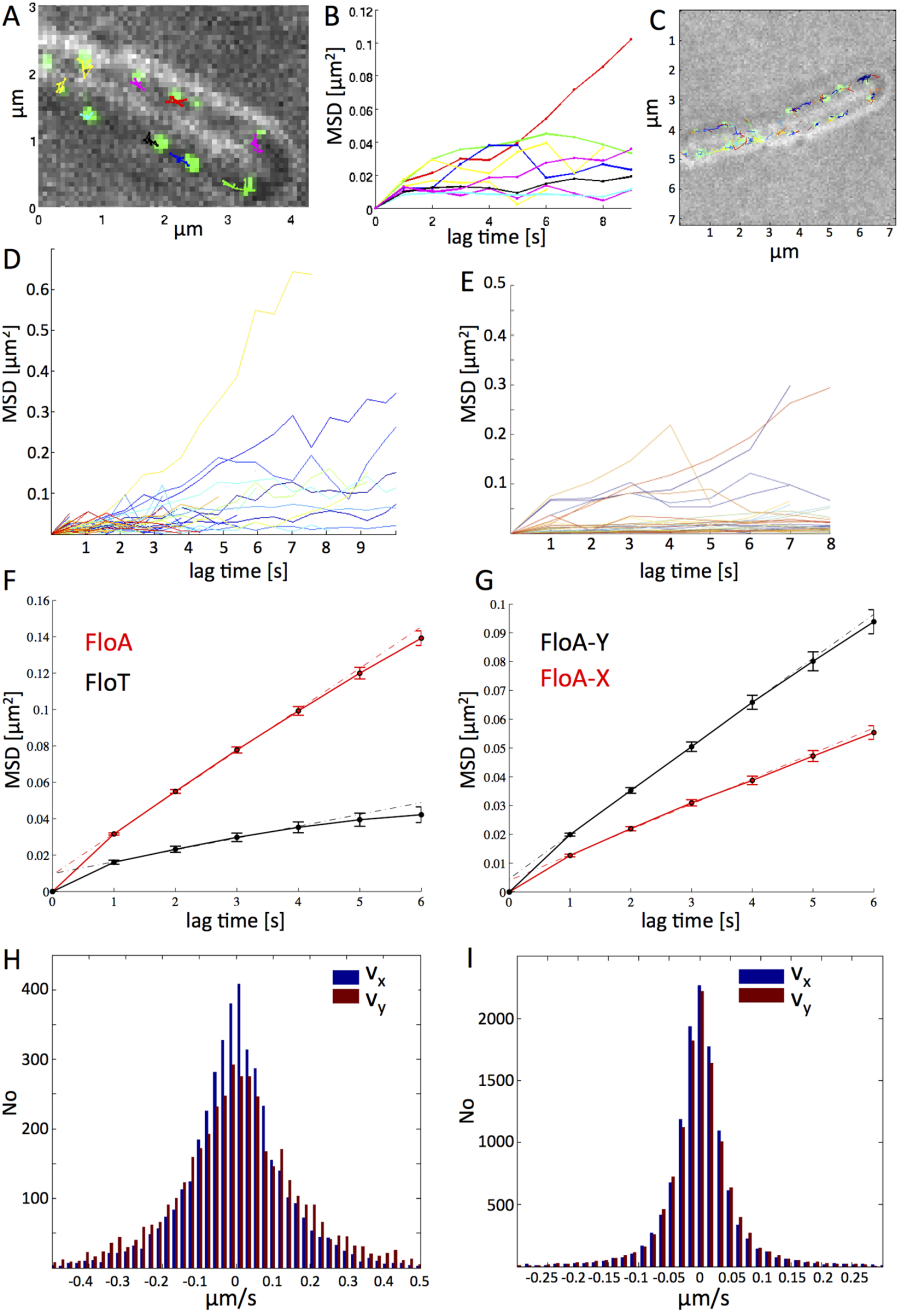
Dynamics of *Bacillus* flotillin structures. A) overlay of a bright field image of a single *Bacillus* cells with the first frame of fluorescence (green signals) and of 9 trajectories, each having a different colour, of YFP tagged protein FloA, displayed over a time course of 8 seconds. B) individual mean squared displacement curves of the 9 particles observed (in corresponding colours) C) Example of about 50 tracks in 2 *Bacillus* cells, which are analysed in D) MSD curves for FloA; E) MSD curves of FloT for an experiment analogous to that shown in A) and C). F) weighted mean over all tracked MSD curves of FloA (3152 tracks, red curve) and of FloT (N = 1024, black curve). Error bars indicate the confidence of the mean values, (given by the weighted standard deviation, divided by the number of degrees of freedom). Dotted lines show the fits to the diffusion constants. G) Plot like in panel F), but differentiated into movement in the y-direction or in the x-direction for FloA-YFP; H) distribution of instantaneous velocities of FloA and along the perpendicular (v_x_, blue bars) and longitudinal (v_y_, red bars) cell axis, No = number. I) same as H) for FloT. For FloA, the tails of the distribution are visibly broader than for FloT, indicating a higher variance and therefore a higher diffusion constant of FloA.

Movement of flotillin proteins has been described as “dynamic”, however, given exposures with 3 s or 2 min intervals [22, 23], almost every membrane protein is expected to be dynamic, i.e. to move between time points of image acquisition. We therefore performed fast time lapse experiments using 1 s intervals (and 0.3 s exposures), or 0.3 s stream acquisitions. Interestingly, dynamics of FloT and of FloA were clearly distinguishable, in that most FloT-YFP signals moved visibly slower than FloA-YFP signals (compare S2 and S3 Movies), and that more static FloT-YFP foci than for FloA-YFP were present in cells. In order to quantify the differences in movement, we automatically tracked and analyzed flotillin foci using Trackmate (ImageJ plugin) software. Fig 5A shows an example of 9 FloA-YFP tracks, overlaid with the first frame of the acquisition, in a single cell, and Fig 5B shows the velocity of the 9 trajectories over time. We used this strategy with a total of 3152 FloA Tracks and 1024 for FloT. Fig 5C shows 2 cells with about 50 trajectories, whose mean squared displacements (MSDs) are plotted in Fig 5D, revealing that most FloA tracks were dynamic, and only a minority was static. A comparison of velocities between FloA (Fig 5D) and FloT (Fig 5E) clearly shows that more static FloT-YFP foci are present in cells, and that FloA-YFP foci in general showed a higher degree of movement.

To gain more insight into the nature of flotillin movement, we determined their diffusion rates. This was done by applying a weighted linear fit to the ensemble mean of all tracked MSD curves of FloA and FloT (Fig 5F). Weights were chosen to take the number of measurement points for each delay time. It is evident that the diffusion of FloA is faster than that of FloT: a fit to the curves revealed a mean diffusion rate of 0.0056 μm^2^/s for FloA-YFP, and 0.0018 μm^2^/s for FloT-YFP. These data show that FloA is about three times as fast as FloT, while both proteins are factor 3 or 9 slower than the large (500 kDa) Tar receptor of chemotaxis, and factor 3.5 to 5 fold (11 to 16 fold for FloT) slower than two other 190 kDa membrane proteins [40]. These data strongly support the idea that flotillins form large assemblies within and at the membrane, which move at considerably different speeds.

As a complication in the tracking of membrane proteins, we considered that the speed of movement of molecules along the circumference of a rod-shaped cell (called “x-axis”) is underestimated because of the projection on the observation plane. Molecules moving along the length of the cell (“y-axis”) move in absolute correlation with the tracks detected by the camera, while molecules moving in “x”-direction move along a curved surface, and thus travel a longer distance per time than detected by the observer (camera). In order to quantify the difference in movement of “X” versus”Y”, we determined MSDs for molecules solely moving in “x” or “y” direction for several frames. A plot of MSD over time shows that apparent velocities along the x-axis are considerably smaller than those along the y-axis, for both proteins (Fig 5G and S4B Fig). Average projected MSD values for FloA-YFP for x-axial movement was 0.0044 μm^2^/s, while that for movement in y-direction was 0.0076 μm^2^/s, and thus about 1.7 fold slower. For FloT, movement in x was 0.0014 μm^2^/s, and 0.0024 μm^2^/s in y, so likewise 1.7 fold lower for x than for y.

To use a different approach for the quantification of diffusion rates, we scored the distances travelled between two time points for foci, which yields a plot for the instantaneous velocity (one time lapse displacement) distribution. Fig 5I shows a much more narrow distribution of FloT-YFP movement compared with that of FloA-YFP (Fig 5H). For FloA, the tails of the distribution are visibly broader than for FloT, indicating a higher variance and therefore a higher diffusion constant of FloA. Diffusion constants can be determined using an unbiased estimator based on the covariance of single time step displacements [var(v) = 2*D/dt]. We can calculate a diffusion constant of 0.0069 μm^2^/s for FloA, and 0.0041 μm^2^/s for FloT; here, FloA is less than 2 fold faster than FloT. For each analysis, the distribution of instantaneous velocities of FloA and FloT was also determined along the minor (v_x_) and major (v_y_) cell axis. In both plots (Fig 5H and 5I), it is apparent that molecules moving along the x-axis move slower (centre more around slower movement), while “y”-tracks can reach much higher speeds. For FloA-YFP, this yields D_x_ = 0.0057 μm^2^/s, and D_y_ = 0.0092 μm^2^/s. Values for FloT are about two fold lower: D_x_ = 0.0028 μm^2^/s, D_y_ = 0.0051 μm^2^/s. Thus, MSDs are 1.6 fold to 1.8 fold higher for the y-axis than for x-axis, and again two fold higher for FloA than for FloT, in good agreement with the MSD values determined through the weighted fit of the MSD curves. Given that molecules travelling in a random fashion along the membrane of a cylinder will travel a mixture of 50% in x and 50% in y direction, our analysis can be used to correct tracks of membrane proteins along a tubular bacterium by multiplying all tracks by factor 1.35, as half of the tracks are underestimated by factor 1.7.

Our experiments rule out the existence of a common structure formed by the flotillin paralogs. The considerably different diffusion rates are not compatible with an interaction of flotillins for more than few milliseconds.

### Flotillin structures can meet and cover the *Bacillus* surface with their movement in a time frame of 1 to 1.5 minutes

With the size of flotillin assemblies at hand and their determined diffusion rate, we can quantify the likelihood of an encounter of the flotillin assemblies within the membrane, which may then exchange interaction partners. Alternatively, if flotillin assemblies are platforms for the organization of membrane proteins that have been inserted and are then released to diffuse by themselves (e.g. KinC), we can estimate the time it takes for assemblies to cover the membrane surface via random movement. Towards this end, we quantified the number of FloT-YFP and of FloA-YFP assemblies in 50 cells, using deconvoluted G-STED images. During exponential growth, cells contained 8.6 ± 2.2 (SD, n = 50) foci of FloT on one side of the cell, and 12.3 ± 4.1 (SD, n = 50) of FloA. Thus, in total, cells have 17 FloT and 25 FloA assemblies on their membrane surface.

The surface of Bacillus is about 9 μm^2^, given that cells are on average 3 μm long (2 to 4 μm) and 1 μm thick (leaving out the polar regions). If the surface is divided by the number of proteins, each FloA assembly will have an area of 0.36 μm^2^ for itself, or a square with a side length of 0.6 μm. Given a diffusion constant of (average of the covariance method and the MSD fit times 1.35) 0.0084 μm^2^/s, the protein will need about 43 s on average, in order to meet another FloA assembly. The size of 80 nm for FloA assemblies means that their size is of minor importance, because on average, there are 600 nm of space between FloA assemblies that need to be bridged via random diffusion for a much smaller sized object. This would change considerably if flotillin assemblies had a larger size, e.g. closer to the diffraction limit of conventional light microscopy (250 nm); such large assemblies would “meet” much more often. In other words, it will take about three quarters of a minute for all FloA assemblies to largely cover the surface of a *Bacillus* based on their diffusion. For FloT (average corrected diffusion rate 0.004 μm^2^/s), the average time to “meet” is accordingly 90 s. A static membrane-associated protein would thus be picked up by a flotillin T assembly in one and a half minutes on average.

### FloT forms a common protein domain with NfeD2, but not with FloA or with other DRM-associated proteins

**Fig 6:**
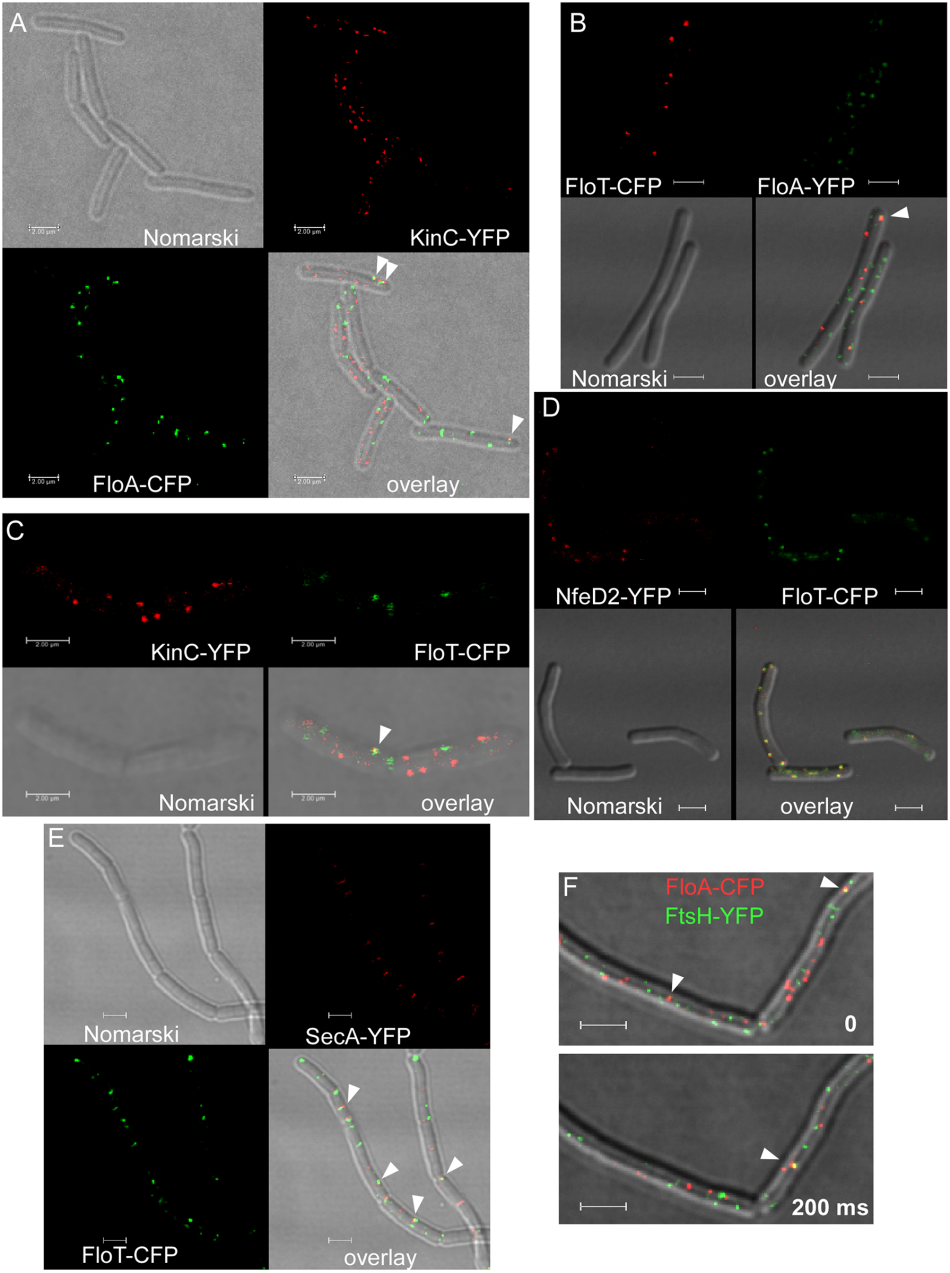
Localization of flotillins and associated protein in exponentially growing *B*. *subtilis* cells expressing two protein fusions. A) FloA-CFP and KinC-YFP, Nomarski-DIC, overlay of Nomarski-DIC, FloA-CFP (green) and KinC-YFP. B) FloT-CFP, FloA-YFP, overlay of Nomarski-DIC and FloT-CFP (red) and FloA-YFP (green). C) KinC-YFP, FloT-CFP, overlay of Nomarski-DIC and KinC-YFP (red) and FloT-CFP (green). D) NfeD2-YFP and FloT-CFP, overlay of Nomarski-DIC and NfeD2-YFP (red) and FloT-CFP (green). E) SecA-YFP and FloT-CFP, overlay of Nomarski-DIC and SecA-YFP (red) and FloT-CFP (green), F) overlay of FtsH-YFP (green) and FloA-CFP (red), images of the two panels were taken with an interval of 200 ms. White triangles indicate cases of colocalization, white bars 2 μm.

KinC plays an important role in signal transduction during cell differentiation and biofilm formation, and has been shown to colocalize with FloT and with FloA [26]. A C-terminal fusion to KinC has been shown to support all known functions of the protein [26]. When imaged in G-STED, KinC-YFP formed foci with an average diameter of 68.7 nm ± 6.6 nm (SD) (Fig 1F). Based on the finding that KinC-YFP foci disappear after the loss of FloA and of FloT, and were proposed to colocalize with flotillins [26], these data suggest that KinC is part of the 70 nm FloT and/or FloA structures. However, when we imaged KinC-YFP together with FloA-CFP or with FloT-CFP (expressed at low level from an ectopic site on the chromosome, such that the average number of foci did not increase), even using conventional confocal microscopy and deconvolution (for the removal of background fluorescence), we found only a minor degree of colocalization. We took advantage of the “between lines” acquisition mode during confocal microscopy, in which each line is first scanned with one laser line and then with the second, before moving to the next line, thereby avoiding a drift of signals between the acquisition of the two channels. Lines are scanned with 400 Hz, i.e. the interval between channel acquisition is 2.5 ms. Our experiments clearly establish that KinC rarely colocalized with FloA: only 3% of the signals showed an overlap (Fig 6A, S5 Fig, 220 cells analysed). Colocalization with FloT was 4% (Fig 6C, S5 Fig, 200 cells analysed), indicating that more than 90% of KinC signals do not colocalize with any flotillin, given that FloT and FloA do not colocalize [23], which can be seen in Fig 6B (less than 2% colocalization). Therefore, flotillins have a similar size as KinC assemblies, but cannot be the architectural basis of the latter. Our data clearly rule out that flotillins and KinC are part of the same “microdomain” structures, and show that flotillins do not have an appreciable spatial overlap, as has been proposed before [27, 28].

We analysed a second protein, NfeD2, that has been described to colocalize with flotillins (FloT), and whose localization into discrete foci depends on FloT [23]. *NfeD2* is cotranscribed with *floT* (*yuaG*), which is a common feature of NfeD encoding genes. NfeD2-YFP also formed defined foci, however, with an average size of 94.9 nm ± 10.1 nm (SD) (Fig 1G), which are considerably larger than the structures of its associated partner, FloT. In the absence of FloT, no defined NfeD2-YFP assemblies were visible, but only delocalized fluorescence throughout the membrane was detectable [23]. On the other hand, more than 96% of FloT-YFP and NfeD2-CFP colocalized (Fig 6D, S5 Fig), and consequently, NfeD2 must be part of the (85 nm sized) assemblies of FloT. Given the larger size of NfeD2 assemblies, it appears that the protein forms an outer “rim” around the FloT structure. We also tested the effect of a deletion of *nfed2* on the localization of FloT-YFP. Although the visual impression of FloT-YFP foci in epifluorescence was that of more foci with less intensity [23], FloT-YFP foci still had an average size of 85 nm (S5C Fig). Thus, the absence of NfeD2 has no visible effect on the structure of FloT assemblies. We wondered if NfeD2 provides an important function for the activity of FloT assemblies. An *nfeD2* deletion has no discernable phenotype [23]. To test if the lack of NfeD2 has a cryptic effect, we generated a *floA nfeD2* double mutant strain, because the absence of FloA and FloT leads to a strong phenotype, in contrast to single *floA*, *floT* or *nfeD2* deletions [23]. However, *floA nfeD2* double mutant cells did not reveal any discernable defect in growth or in cell morphology (S5E Fig), revealing that NfeD2 is not essential for the known functions of FloT (i.e. it does not impair the functions that lead to the synthetic phenotype with FloA). In any event, NfeD2 is a clear example of a protein that colocalizes with a flotillin, showing that the rare flotillin/KinC co-localization is not a technical artifact.

Based on the different dynamics of flotillin paralogs, we investigated the putative interaction partners NfeD2-YFP and KinC-YFP using time lapse microscopy. While NfeD2-YFP showed similarly slower movement like FloT (S5 Movie), KinC-YFP signals moved much faster than FloT assemblies (S4 Movie). Thus, NfeD2 will track with FloT, because of the major degree of colocalization of the two, while KinC will not track with FloT, because of the different dynamics, reinforcing the non-significant colocalization between the proteins.

The lack of colocalization between flotillins and KinC prompted us to perform dual colour imaging with further proteins that have been described to be co-isolated with FloT and/or FloA, and to co-localize with flotillins based on epifluorescence microscopy [27, 28]. We chose FtsH and SecA, because both proteins perform vital functions for the physiology of the cell, and because both proteins have been described to belong to the DRM fraction. Interestingly, the absence of both, FloT and FloA has been shown to lead to a depletion of FtsH from the cell membrane [28]. We used FP fusions that have been shown to functionally replace the wild type proteins [27, 28, 41], and which also in our hands do not show a discernable phenotype when expressed as sole copy from the original gene locus. S6A Fig shows that there is some degree of colocalization between FloT-CFP and FtsH-YFP (5% of foci), and between FloA-CFP and FtsH-YFP (Fig 6F, S6C Fig, 6% colocalization). Flotillins and FtsH were frequently found to be present at sites of cell division (S6A and S6B Fig), in agreement with the findings that both types of proteins play an important role in cell division [23, 42]. These experiments suggest that the vast majority of flotillin assemblies do not form a common structural assembly with FtsH. As a control, we analysed if FloT would colocalize with itself, using two FloT alleles that are fused to CFP or to YFP, respectively. S6F Fig shows that both populations mixed, to yield more than 90% colocalization. We tested three further proteins that were reported to interact with flotillins: histidine kinases PhoR and ResE, and oligopeptide permease OppA [43]. S7 Fig shows representative images of dual colour experiments. 4.5% of OppA-YFP foci colocalized with FloT-CFP (S7A Fig, 118 cells analysed), 6% of RecE (S7B Fig, 90 cells analysed) and 7% of PhoR-YFP (S7D Fig, 78 cells analysed each). 12% of PhoR-YFP signals colocalized with FloA-CFP (S7C Fig, 43 cells analysed). These data show that flotillins colocalize with other membrane proteins in few cases, and the majority of flotillin domains do not contain any visible numbers of membrane proteins we have tested.

### DRM proteins move through the membrane along different paths

To further support the finding that flotillins and other DRM proteins form distinct microdomains, and to investigate if transient interactions exist between DRM proteins, we performed two colour time lapse experiments. Joint formation of protein microdomains would mean co-migration of signals, while absence of joint movement would agree with the existence of generally separate entities. Fig 6F shows two intervals of such an experiment, where a colocalization event between FloA and FtsH (upper panel) no longer exists after 200 ms (lower panel), while in the second interval, a colocalization can be seen at a different place in the cell. S6 Movie shows that the overwhelming majority of signals do not move together, in agreement with the low degree of colocalization seen in the snap shot images. Similarly, FloT-CFP and FtsH-YFP did not move together in a detectable manner (S7 Movie), and in agreement with a lack of colocalization, FloT and KinC did not show movement of common domains between 200 ms acquisitions (S8 Movie).

The highest degree of colocalization was seen for FloT-CFP and SecA-YFP (22% of the foci, Fig 6E), and between FloA-CFP and SecA-YFP (15%, S6D Fig). However, also in this case, a majority of foci did not colocalize, showing that flotillin T and SecA do not generally form mixed protein structures. We also performed dual colour time lapse microscopy, revealing that the majority of FloT-CFP and SecA-YFP foci moved independently from each other (S9 Movie). A significant number of foci coincided during image acquisition, but we never detected co-migration for more than 2 frames. Thus, FloT and SecA interactions also take place in the sub-second range. It should be noted that colocalization does not necessarily mean interaction, because the protein domains may be within a range of 250 nm (resolution of conventional light microscopy) but not completely coincide based on their size of less than 100 nm. As such, the numbers determined in our analysis are an overestimate of possible interactions. Contrarily, FloT-CFP signals invariably moved together with NfeD2-YFP signals (S10 Movie), while FloT and FloA did not track together as expected (S11 Movie).

### DRM proteins move through the membrane with different velocities

**Fig 7:**
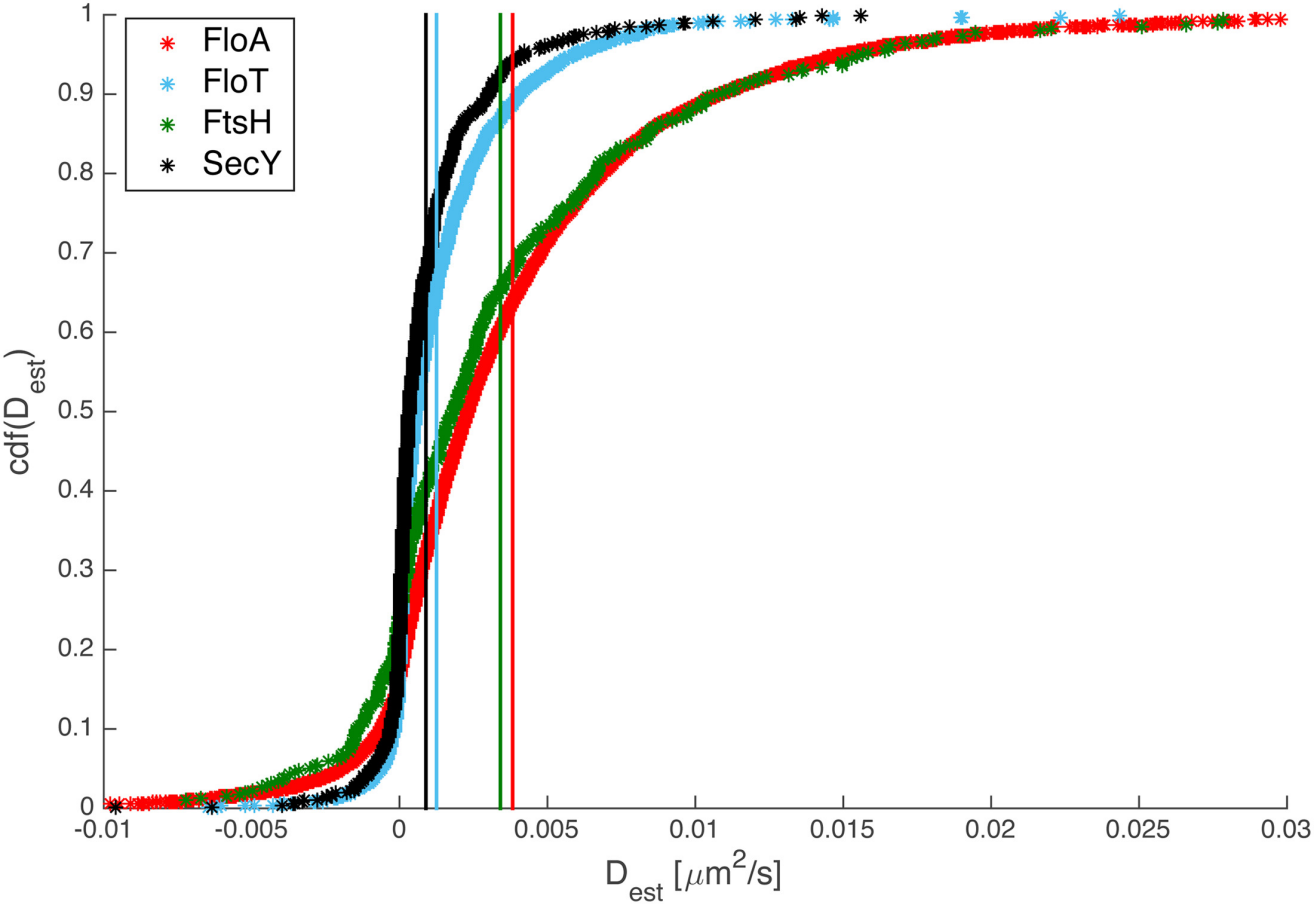
Cumulative probability distributions of diffusion coefficients for different proteins. The diffusion coefficient for each observed track is calculated using a covariance based estimator (CVE) as proposed in [48] and the empirical cumulative probability distribution of the resulting coefficients is calculated. The distribution of FloT shows a steep rise at small diffusion coefficients, indicating slower dynamics compared to FloA. Vertical lines mark the mean value of each distribution.

We automatically tracked FloA-YFP, FloT-YFP, FtsH-YFP and SecA-YFP, and compared their dynamics using cumulative probability distributions of diffusion coefficients. Fig 7 shows that FloA, FloT and FtsH had clearly distinguishable dynamics, resulting in different diffusion constants as indicated by the vertical lines. Note that the small number of negative diffusion coefficients occur due to the statistical nature of the covariance based estimator (CVE). SecA interacts with the SecYEG translocon, and shows very similar dynamics as FloT. However, based on the colocalization experiments, this cannot be taken as an argument that the proteins move together in a common microdomain. In agreement with the basal level of colocalization of FloA, FloT and of FtsH, and between FloA and SecA, the different diffusion kinetics show that each proteins moves with a different characteristic speed through the membrane.

These experiments validate our findings that flotillins do not form joint microdomains with proteins that have been co-isolated by special detergent conditions (KinC, FtsH, SecA, ResE, PhoR or OppA) for more than few milliseconds, supporting the idea that FloT forms a common structure with NfeD2 (many flotillin genes lie adjacent to genes coding for NfeD proteins), and suggest a close connection between flotillins and the Sec system compared with other tested proteins.

## Discussion

Membranes in eukaryotic cells have been shown to contain asymmetries in lipid composition, and to contain nanoscale, cholesterol-assisted, dynamic and selective protein assemblies, which can coalesce into larger and more permanent “raft” structures [44]. Such mixed protein domains have been shown to play important roles in TCR signalling, HIV assembly, endoplasmic reticulum (ER)-to-Golgi and post- Golgi trafficking to the cell surface, and glycosphingolipid mediated endocytosis. In bacteria, a variety of membrane proteins has been reported to form visible clusters in the cell membrane, and the existence of raft structures or functional microdomains has been deduced from the finding that a fraction of Triton-insoluble proteins exists (so called detergent resistant microdomains, DRMs), which can be co-isolated with flotillins [27, 28]. The latter protein family is conserved between bacteria and eukaryotes, is found in the DRM fraction in both cell types, and is associated with raft structures in eukaryotic cells, where it is involved in the clustering of membrane proteins and in various aspects of membrane trafficking [8, 45]. Our study was performed to determine the actual size of flotillin and DRM protein clusters in living cells, to investigate the dynamics of clusters within the membrane, and to investigate if the two flotillin proteins in *B*. *subtilis*, FloA and FloT form joint clusters with other DRM proteins.

A major conclusion from our *in vivo* analyses is the finding that protein clusters formed by flotillins in bacteria, in a fungus and in human cells have a relatively defined size of 85 to 110 nm, and that likewise, DRM protein KinC forms structures of a similar diameter. However, FloT and FloA do not colocalize with each other, and colocalize only with a minority of FtsH, SecA or KinC assemblies. These findings are substantiated by the determination of the movement of bacterial flotillins, whose dynamics are different by a factor of 2, revealing that the proteins do not track together. Time lapse experiments show that FloT or FloA assemblies do not co-migrate with any of the other investigated proteins, the exception being the co-migration of FloT and NfeD2, which shows a tight connection for these two proteins, in agreement with a conserved connection of the genes in many organisms [9]. Therefore, our study reveals the existence of protein domains in the sub-100 nm range, which show independent movement, and rule out the generation of large multiprotein clusters generated by flotillins, at least in case of the investigated proteins. Co-localization events between flotillins and other membrane proteins are observed for 200 ms intervals, showing that putative interactions between the domains are highly dynamic and transient; it must be noted that the domains may not even physically interact but merely move past each other. Therefore, integral membrane proteins that are co-isolated based on insolubility to cold Triton extraction do not necessarily form joint microdomains, but assemble into largely distinct multiprotein clusters of a characteristic size. It should be noted, that our confocal and epifluorescence microscopy experiments visualize bulk protein, not individual molecules. It is therefore possible, that flotillin microdomains contain few molecules of other proteins. In other words, our analyses do not rule out that a fraction of a given protein can be found within different microdomains, and that proteins may partition into different membrane domains based on diverse affinities.

Interestingly, the size of flotillin clusters was relatively robust against changes in specific lipid environment of the proteins. Under conditions of low levels of cardiolipin, lack of phosphatidyl-ethanolamine, of lysylphosphatidyl-glycerol or of glycolipids, we still observed the generation of 100 nm flotillin domains, suggesting that protein/protein interactions play a major role in the formation of these structures. This is corroborated by our *in vitro* studies in which purified soluble FloA forms up to 70 nm large structures visualized by electron microscopy, indicating that the assembly of flotillin structures is largely driven by protein/protein interactions. The apparent robustness of microdomain formation against lipid perturbations notwithstanding, it is possible that flotillins themselves organize a specific lipid microenvironment that is important for their as yet unknown function. It will be important to determine in how far lipids affect the formation of assemblies of other membrane proteins.

Flotillin/reggie proteins are involved in several membrane-dynamics in eukaryotic cells, and play an important role in the physiology of bacteria. Absence of both flotillins from *B*. *subtilis* leads to compromised growth, loss of proper cell shape and a defect in cell division, besides reduction in motility [23, 30]. None of these severe phenotypes are observed in the absence of a single flotillin, which has been used to support the idea that the two proteins form joint raft structures and recruit e.g. signaling molecules to the microdomains. Our results clearly show that the important function(s) of flotillins is mediated by two independently localizing and moving fractions, and that therefore, the two paralogs are functionally distinguishable.

FloT recruits NfeD2 into a joint assembly, suggesting that the two proteins cooperate in these assemblies, however, we show that the presence of NfeD2 does not have a strong influence on the function of FloT, based on the lack of any phenotype in the absence of FloA and of NfeD2. Multimerization is inherent to flotillins, based on the assembly of the soluble part of FloA into large oligomers *in vitro*, which are rather stable. Although there is a dynamic exchange of flotillin multimers and the smallest subunits formed (most likely tetramers), the equilibrium lies far on the side of polymer formation. Determination of subunit stoichiometry of FloT-YFP in living cells showed that the assemblies consist of at least 12 monomers, whose exchange occurs in a range of seconds. The diffusion of flotillins, especially of FloT, is very slow compared with other bacterial membrane proteins, in agreement with the large number of flotillin monomers within the assemblies. Based on our calculation it takes flotillins between 45 to 90 seconds to “scan” the entire surface of a rod shaped bacterium. Therefore, it is likely that flotillins respond rapidly to their substrates or binding partners, for example assuming that flotillins aid in the insertion of proteins such as FtsH into the membrane [28].

We show that the movement of molecules on the curved surface of a rod-shaped organism differs dependent on the direction of movement, which is underestimated by a factor of 1.7 in case of *B*. *subtilis* (diameter of 1 μm) when molecules move perpendicular to the long cell axis (“x”) compared to the longitudinal movement (“y”). As on average, molecules move 50% in x and 50% in y direction in case of random diffusion (which is the case for flotillins and for all DRM proteins), a factor of 1.35 can be used to correct for the curvature-caused underestimation of diffusion. This calculation can be used for all cells having a similar diameter as *B*. *subtilis*, and can be adapted to other cell diameters for accurate calculations of protein dynamics. Our calculation that flotillin microdomains meet in the range of seconds is an important tool to determine interaction kinetics of protein microdomains within the cell membrane in bacteria. An analogous function concerning the activity of eukaryotic flotillins, i.e. the targeted delivery of bulk membrane and specific membrane proteins from internal vesicle pools to strategically important sites has been suggested [8].

If bacterial flotillins do not act as a molecular scaffold within the membrane, what may be their function? In the absence of flotillins, cells suffer from many defects, such as membrane abnormalities, loss of proper cell shape, defects in cell division and in motility, and a delay in sporulation [22, 23, 30]. We propose a model in which bacterial flotillins act as membrane insertion-helpers for membrane proteins that require a special lipid environment. We suggest that flotillins transiently interact with the secretion machinery during the insertion of e.g. KinC and FtsH. This is supported by our finding that FloT shows the strongest degree of colocalization with SecA, the central component of the secretion machinery. Also, the depletion of FloT and of FloA has been shown to lead to a strong reduction of the amount of FtsH within the membrane [28], which is most easily explained through a defect in insertion, unless FtsH becomes more prone to degradation in the absence of flotillins. Since there is a large number of FtsH assemblies that does not colocalize or co-migrate with flotillins, the latter scenario seems rather unlikely. With regard to their different diffusion rates, FloT would be engaged in membrane insertion for longer periods of time, while FloA would be quicker and more abundant to meet with insertion islands.

Tracking of flotillins, of FtsH and of SecA demonstrated distinct diffusion kinetics for each protein. Therefore, our data reveal the existence of protein domains consisting of clusters of the same protein, which in case of flotillins, KinC, SecA and FtsH do not overlap, and are only transiently in close proximity enabling an interaction, which can take place in the range of few hundred milliseconds. Our findings have a profound impact on our view of the mode of movement and interaction of membrane proteins and of the organization of DRM proteins. It will be important to investigate the overall movement of many membrane proteins to obtain a clear view on the two dimensional organization and dynamics of membrane proteins.

## Materials and Methods

### Construction of strains

For expression of soluble 6xHis-FloA, the coding sequence lacking the first 10 codons was amplified by PCR using chromosomal DNA from the *B*. *subtilis* wild type strain PY79. The fragment was further integrated in the expression vector pET24d (Novagen) by *Nco*I and *Bam*HI restriction ligation and brought into the expression host *E*. *coli* BL 21 (DE3) giving rise to the strain FD380. For expression of soluble Strep-FloA, the coding sequence (minus the first 10 codons) was amplified by PCR (the upstream primer contains the Strep tag sequence) using chromosomal DNA from *B*. *subtilis* PY79. The fragment was inserted into pET24d by *Nco*I and *Bam*HI restriction ligation and the resulting plasmid was introduced into the expression strain *E*. *coli* BL21 (DE3), yielding strain AHV11.

KinC was visualized as a KinC-YFP fusion protein expressed at the original locus. The last 500 bp coding for *kinC* were integrated into the vector pSG1164-YFP, using *Apa*I and *EcoR*I restriction sites, and PY79 cells were transformed with this construct, selecting for cm resistance (leading to strain FD326). For colocalization studies, *floA-cfp* was integrated at *amyE* locus (by the use of the plasmid pSG1192 [46] and expression was controlled by xylose addition. The resulting strain (AS34, *floA-cfp*::*amyE*^specR^) was transformed with chromosomal DNA of strain FD326 leading to a strain AS38 expressing KinC-YFP and FloA-CFP. To investigate colocalization of KinC and FloT, *floT-cfp* was integrated at the *amyE* locus under the control of the P_xyl_ promoter in the strain FD326, giving rise to AS49. For SecA and FtsH, analogous strategies were used. 500 bp of the 3’ end of each gene were integrated into pSG1164, using *Apa*I and *Eco*RI sites, and the resulting strains were transformed with chromosomal DNA from strains AS34 (*floA-cfp*::amyE^specR^) or from AS49 (*kinC-yfp*^catR^ *floT-cfp*::amyE^specR^), selecting for spec resistance. Similarly, for the colocalization of OppA, of ResE or PhoR with FloT or with FloA, chromosomal integrants were generated expressing OppA-YFP, ResE-YFP or PhoR-YFP, and were transformed with DNA from the strains expressing FloA-CFP or FloT-CFP from the amylase locus.

For the generation of lipid mutant strains expressing FloA-YFP or FloT-YFP, strain 168 containing either deletions in *clsA*, or in *pssA*, or in *ugtP*, or in *mprF*, were transformed with chromosomal DNA from strain AS34 (*floA-cfp*::*amyE*^specR^) or from strain AS49 (*kinC-yfp*^catR^ *floT-cfp*::*amyE*^specR^), selecting for spectinomycin resistance. As control strain, *B*. *subtilis* 168 was transformed with chromosomal DNA from strains FD191 (*floA-yfp*^catR^) or from FD295 (*floT-yfp*^catR^), giving rise to FloA-YFP or FloT-YFP expressing 168 wild type strains. All strains are listed in Table 2.

**Table 2:** 

Strain	Genotype	Reference
PY79	Wild type	
DH5α	*fhuA2 Δ(argF-lacZ)U169 phoA glnV44 Φ80 Δ(lacZ)M15 gyrA96 recA1 relA1 endA1 thi-1 hsdR1*	NEB
BL21	*fhuA2 [lon] ompT gal (λ DE3) [dcm] ΔhsdS*	New England Biolabs (NEB)
FD380	BL21 pET24d 6his-floA_sol_	This study
AHV11	BL21 pET24d strep-floA_sol_	This study
FD326	*kinC-yfp*^catR^	This study
FD191	*floA-yfp*^catR^	[29]
FD295	*floT-yfp*^catR^	[29]
FD350	*floT-cfp*^catR^ *yuaF-yfp*^catR::tetR^	[29]
FD124	*nfeD2-yfp*^catrR^	[29]
SNT122	*floA-gfp*	[19]
AS34	*amyE*::*psg1192 floA-cfp*^specR^	This study
AS49	*kinC-yfp*^catR^ *floT-cfp*^specR^	This study
AS38	*kinC-yfp*^catR^ *floA-cfp*^specR^	This study
FD175	*floT-yfp*^catR^ *yuaF*::*SpC*	[29]
AS66	*floT-cfp*^catR^ *secA-yfp*^catR::tetR^	This study
AS61	*secA-yfp*	This study
AS51	*ftsH-YFP*	This study
AS53	*floT-cfp*^catR^ *ftsH-yfp*^catR::tetR^	This study
AS52	*floA-cfp*^catR^ *ftsH-yfp*^catR::tetR^	This study
AS56	*floA-cfp*^catR^ *floT-yfp*^specR^	This study
FD506	*floT-yfp*^*catR*^, *floT-cfp*^*specR*^	This study
FD344	*floT-cfp*^catR^ *floA-yfp*^catR::tetR^	[29]
168	*trpC2*	Laboratory strain
HB5346	*ugtP*::*MLS*	[34]
HB5347	*clsA/ymnE*::*tet*	[34]
HB5361	*pssA*::*spec*	[34]
HB5337	*mprF*::*kan*	[34]
CR1	*ugtP*::*MLS floA-yfp*^*catR*^	This study
CR2	*ugtP*::*MLS floT-yfp*^*catR*^	This study
CR3	*pssA*::*spec floA-yfp*^*catR*^	This study
CR4	*pssA*::*spec floT-yfp*^*catR*^	This study
CR5	*mprF*::*kan floA-yfp*^*catR*^	This study
CR6	*mprF*::*kan floT-yfp*^*catR*^	This study
CR7	*floA-yfp*^*catR*^	This study
CR8	*floT-yfp*^*catR*^	This study
CR9	*clsA/ymnE*::*tet floA-yfp*^*catR*^	This study
CR10	*clsA/ymnE*::*tet floT-yfp*^*catR*^	This study
AHV17	*floT-cfp*^*specR*^, *resE-mVyfp*^*catR*^	This study,
AHV18	*floT-cfp*^*specR*^, *oppA-mVyfp*^*catR*^	This study
AHV19	*floA-cfp*^*specR*^, *phoR-mVyfp*^*catR*^	This study
AHV20	*floT-cfp*^*specR*^, *phoR-mVyfp*^*catR*^	This study

### Cell growth and microscopy

Cells were grown to late exponential or stationary phase in LB rich medium containing the appropriate antibiotics at 37°C and under aeration. Xylose was supplemented to induce expression of proteins downstream of the fusion protein at original locus (0.5% (w/v)) or the fusion protein itself at *amyE* locus (0.01% (w/v)). For microscopy, 2.5 μl of the culture were spotted on a coverslip and immobilized by an agarose pad (1% agarose (w/v) in S7_50_ glucose minimal medium). STED microscopy was performed at a Leica SP8 LSM confocal microscope equipped with a 100X objective (NA 1.4) and a 592 nm depletion laser. Fluorophores were excited with a pulsed white light laser source at 514 or 488 nm respectively. Photon emission was detected with gated hybrid detectors at the appropriate wavelength. Images were processed with the Leika LAS AF software and where stated deconvolution was performed using the Huygens-algorithm (SVI). For colocalization studies, images were acquired simultaneously, by scanning each line first in the YFP (514 nm) than in the CFP (457 nm) channel. Epifluorescence microscopy was done using a Zeiss Axio Imager A1 equipped with an EVOLVE EMCCD camera (Photometrics) and a TIRF objective with an aperture of 1.45, acquiring images with VisiView (2.1.2, Visitron, Munich) software and using a 515 nm laser for YFP detection and a 445 nm laser for CFP detection. For the determination of subunit numbers, a 514 nm diode laser was directly incorporated into the microscope and was focused on the back focal plane, such that a roughly 5 x 5 μm^2^ illumination spot is generated in the focal plane. Images were acquired by frame transfer stream acquisition, with exposure times of 20 ms, by a Hamamatsu Image EM2 EM-CCD camera.

The *A*. *nidulans* strain, SNT122, expresses FloA tagged with GFP at the C-terminus under the native promoter [19]. Spores of the strain were inoculated in minimal medium + 2% glucose (w/v) and were incubated at 30°C overnight.

MCF-7 and HeLa cells were cultured at 37°C and 5% CO_2_ in MEM and DMEM respectively, supplemented with 10% FCS, L-glutamine and penicillin/streptomycin. Transfection was carried out using the lipofectamine 2000 transfection reagent (Life Technologies) following the manufacturer’s instructions and cultured for 48 h on poly-L-lysine coated coverslips. Cells were fixed with 4% PFA and prepared for microscopy using Mowiol (Sigma Aldrich). The Reggie-1-EGFP (flotillin-2) vector was described previously [2].

### Protein tracking and estimation of diffusion coefficients

Protein movement was recorded using time lapse microscopy with a frame rate of 1 frame per second and a faster rate of 3 frames per second. The protein positions were tracked using Trackmate, a plugin of the image analysis software ImageJ. The resulting tracks were corrected for drift, to account for movement of the whole cell during acquisition.

To distinguish between movement along (y—direction) and normal (x—directions) to the cell axis, the position and orientation of each observed cell was recorded and corresponding protein tracks were transformed into the coordinate system of the cell.

Diffusion coefficients were obtained using a weighted linear fit of the ensemble mean square displacement curve. Since data points in the MSD curve are highly correlated [47], only the first three points were used in the fit. A second method based on the analysis of the distribution of one time lapse displacements [48] was applied to confirm the results obtained by MSD analysis.

### Protein purification

The soluble part of FloA (amino acids 56–331 complemented with a C-terminal Trp residue for better visibility at 280 nm) was first C-terminally fused to a 6xHis tag in the pET24d vector (Novagen) and purified by Ni-NTA affinity chromatography. The protein was heterologously expressed in *E*. *coli* BL21 (DE3) cells overnight at 30°C by addition of lactose (1.75% (w/v)), cells were collected by centrifugation and the pellet was resuspended in 30 ml of buffer A (20 mM HEPES, 250 mM NaCl, 20 mM MgCl_2_, 20 mM KCl, 40 mM imidazole, pH 8). Then the cells were broken in a Microfluidizer (Microfluidics) and the soluble and insoluble fractions were separated by centrifugation (30 min at 30000 g at 4°C). The supernatant was loaded onto a His-Trap column (GE Healthcare), washed with buffer A and the protein eluted with buffer B (buffer A with 500 mM imidazole).

For the analysis without His-tag, the soluble part of FloA was C-terminally fused to a Strep-tag (substituting the His-tag sequence by the Strep-tag sequence in pET24d) and purified by Strep-Tactin affinity chromatography. An *E*. *coli* BL21 (DE3) strain harboring the pET24d-floAstrep plasmid was grown in LB media at 37°C until mid-exponential phase. Then the overexpression of FloAstrep was induced by the addition of 1 mM IPTG to the culture and incubated for 2 h at 37°C. Cells were collected by centrifugation, the pellet was resuspended in 40 ml of TE-based buffer (100 mM Tris-Cl, 1 mM EDTA, 150 mM NaCl, pH 8) and the cells were broken by passage through a French Press. The soluble fraction was obtained by centrifugation (30 min at 16000 rpm and 4°C) and loaded onto a Strep-Tactin column (Thermo, IBA). The column was washed with TE buffer with 150 mM, 300 mM and 500 mM NaCl and the protein eluted with elution buffer (100 mM Tris-Cl, 1 mM EDTA, 150 mM NaCl, 2.5 M D-Desthiobiotin, pH 8).

For further purification and analysis of oligomerization, both FloA6His and FloAstrep were subjected to size exclusion chromatography. The FloA6His protein was loaded in a HiLoad 26/600 Superdex 200 pg column and run at 1.5 ml/min in an HEPES-based buffer (20 mM HEPES, 200 mM NaCl, 20 mM MgCl_2_, 20 mM KCl, pH 7.5), while the FloAstrep protein was loaded in a Superose 6 10/300 GL column and run at 0.5 ml/min in Tris buffer (100 mM Tris-Cl, 150 mM NaCl, pH 7.5).

Folding was analyzed by CD-spectrometry and oligomerization was also determined by a sucrose density gradient (5–20% (w/v)) ultracentrifugation (32500 rpm 4°C, 20 h, rotor SW40Ti, Beckman Coulter Optima XPN-80 centrifuge).

### Electron microscopy

Carbon coated copper grids (400 mesh) were hydrophilized by glow discharging (PELCO easiGlow, Ted Pella, USA). 5 μl of a protein suspension with a concentration of 15 μg/ml was applied onto the hydrophilized grids and stained with 2% uranyl acetate after a short washing step with H_2_Obidest. Samples were analyzed with a JEOL JEM-2100 transmission electron microscope using an acceleration voltage of either 80 or 200 kV. For image acquisition a F214 FastScan CCD camera (TVIPS, Gauting) was used.

## Supporting Information

S1 Movie Single molecule microscopy, stream acquisition (20 ms frames) of cells expressing FloT-YFP as sole source of the protein, 50 frames/s. (AVI)

S2 Movie Exponentially (late stage) growing *B*. *subtilis* cells expressing FloA-YFP from the original gene locus, 400 ms time intervals, shown are 6 frames/s. (AVI)

S3 Movie Exponentially (late stage) growing *B*. *subtilis* cells expressing FloT-YFP from the original gene locus, 400 ms time intervals, shown are 6 frames/s. (AVI)

S4 Movie Exponentially (late stage) growing *B*. *subtilis* cells expressing KinC-YFP from the original gene locus, 400 ms time intervals, shown are 6 frames/s. (AVI)

S5 Movie Exponentially (late stage) growing *B*. *subtilis* cells expressing NfeD2-YFP from the original gene locus, 400 ms time intervals, shown are 6 frames/s. (AVI)

S6 Movie Exponentially (late stage) growing *B*. *subtilis* cells expressing FloA-CFP from the *amy* locus and FtsH-YFP from the original gene locus, 200 ms time intervals, shown are 6 frames/s. (AVI)

S7 Movie Exponentially (late stage) growing *B*. *subtilis* cells expressing FloT-CFP from the *amy* locus and FstH-YFP from the original gene locus, 200 ms time intervals, shown are 6 frames/s. (AVI)

S8 Movie Exponentially (late stage) growing *B*. *subtilis* cells expressing FloT-CFP from the *amy* locus and KinC-YFP from the original gene locus, 200 ms time intervals, shown are 6 frames/s. (AVI)

S9 Movie Exponentially (late stage) growing *B*. *subtilis* cells expressing FloT-CFP from the *amy* locus and SecA-YFP from the original gene locus, 200 ms time intervals, shown are 6 frames/s. (AVI)

S10 Movie Exponentially (late stage) growing *B*. *subtilis* cells expressing FloT-CFP from the *amy* locus and NfeD2-YFP from the original gene locus, 200 ms time intervals, shown are 6 frames/s. (AVI)

S11 Movie Exponentially (late stage) growing *B*. *subtilis* cells expressing FloA-CFP from the *amy* locus and FloT-YFP from the original gene locus, 200 ms time intervals, shown are 6 frames/s. (AVI)

S1 Fig G-STED microscopy of FloA-YFP or of FloT-YFP in different lipid mutant *B*. *subtilis* W168 strains during exponential growth. A) FloA-YFP in wild type cells, B) FloA-YFP in glycolipid mutant cells (Δ*ugtP*), C) FloA-YFP in cardiolipin mutant cells (Δ*clsA*), D) FloA-YFP in lysylphosphatidyl-glycerol mutant cells (Δ*mprF*), E) FloA-YFP in phosphatidyl-ethanolamine cells (Δ*pssA*), F) FloT-YFP in lysylphosphatidyl-glycerol mutant cells (Δ*mprF*), G) FloT-YFP in phosphatidyl-ethanolamine cells (Δ*pssA*), H) FloT-YFP in glycolipid mutant cells (Δ*ugtP*). White bars 2 μm.
(JPG)

S2 Fig Analytical gel filtration (GF) analysis of soluble Strep-FloAsp. A) GF of Strep-FloAsp after Streptavidin affinity chromatography. HMW (high molecular weight) fractions A3 to A8, low molecular weight (LMW) fractions A10 and A11. Fractions in panels showing SDS-PAGE correspond to elution fractions in GF. B) GF of the HMW fraction. Different dilutions of the fraction are marked by different colours. C) GF of the LMW fraction. Because of the low yield of this fraction, it was not diluted but rather concentrated (indicated by different colour lines) to investigate concentration-dependent behavior of fraction formation.
(JPG)

S3 Fig Electron microscopy (negative staining) of the HMW fraction (S2 Fig), showing the different-size particles formed by soluble FloA (JPG)

S4 Fig Particle tracking of FloA-YFP and of FloT-YFP. A) Two examples of FloA-YFP foci (green, overlaid with bright field acquisition) that are tracked over a time of 8 seconds. B) MSD plot for FloT, differentiated into x- and y-movement.
(PPTX)

S5 Fig A) Colocalization of FloT-CFP and NfeD2-YFP, B) Scarce colocalization of FloA-CFP and KinC-YFP, C) localization of FloT-YFP in an *nfeD2* deletion background, D) Scarce colocalization of FloT-CFP and of KinC-YFP. E) Double *floA nfeD2* mutant cells. Colours of foci in overlays correspond to the single channels. Scale bars 2 μm.
(JPG)

S6 Fig Dual colour imaging of B. subtilis cells. A) Confocal microscopy. FloT-CFP rarely colocalizes with FtsH-YFP. The white triangle indicates the presence of a division site, where both proteins frequently localize. B) Chain of cells with several cases of colocalization of both proteins, as seen by the yellow colour in the overlay (indicated by white triangles). Colours of foci in the overlay correspond to the colour of the labelled protein. C) Cases of colocalization of FloA-CFP and FtsH-YFP (white triangles). D) FloA-CFP colocalizes with SecA-YFP in 10 to 20% of the cases (indicated by white triangles). Colours of foci in overlays correspond to the single channels. E) FloT-CFP colocalizes with SecA-YFP in 20% of the foci. F) Colocalization of FloT-YFP expressed from the original gene locus and FloT-CFP expressed from the amylase locus. Scale bars 2 μm.
(JPG)

S7 Fig Dual laser epifluorescence microscopy. Dual colour localization studies of A) FloT-CFP together with OppA-YFP, B) FloT-CFP and ResE-YFP, C) FloA-CFP and PhoR-YFP, D) FloT-CFP and PhoR-YFP. White bars 2 μm.
(JPG)
