# Supplementary figures and images for: Super Resolution Fluorescence Microscopy and Tracking of Bacterial Flotillin (Reggie) Paralogs Provide Evidence for Defined-Sized Protein Microdomains within the Bacterial Membrane but Absence of Clusters Containing Detergent-Resistant Proteins
Source: PLoS Genet. 2016 Jun 30;12(6):e1006116. doi: 10.1371/journal.pgen.1006116 (PMC4928834; doi:10.1371/journal.pgen.1006116)

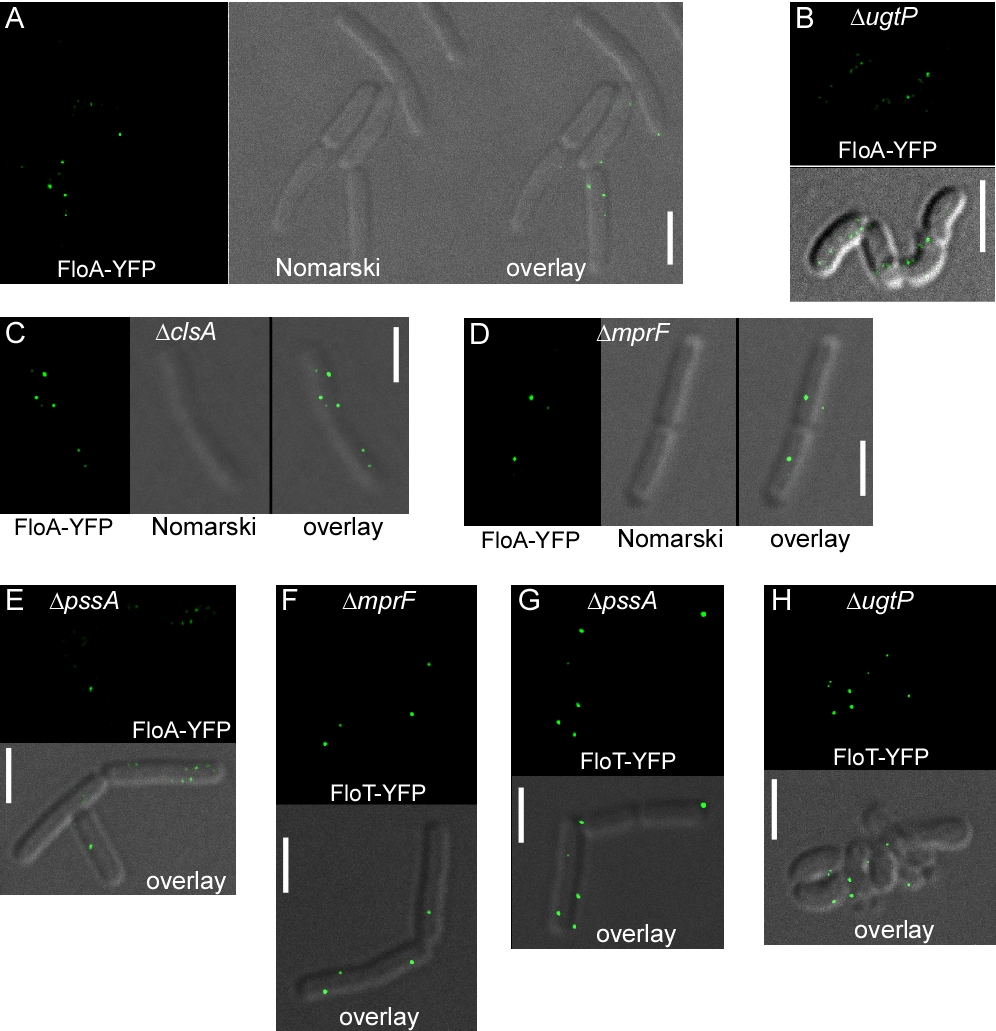

Supplement: S1 Fig — A) FloA-YFP in wild type cells, B) FloA-YFP in glycolipid mutant cells (ΔugtP), C) FloA-YFP in cardiolipin mutant cells (ΔclsA), D) FloA-YFP in lysylphosphatidyl-glycerol mutant cells (ΔmprF), E) FloA-YFP in phosphatidyl-ethanolamine cells (ΔpssA), F) FloT-YFP in lysylphosphatidyl-glycerol mutant cells (ΔmprF), G) FloT-YFP in phosphatidyl-ethanolamine cells (ΔpssA), H) FloT-YFP in glycolipid mutant cells (ΔugtP). White bars 2 μm. (JPG) [file pgen.1006116.s012.jpg]

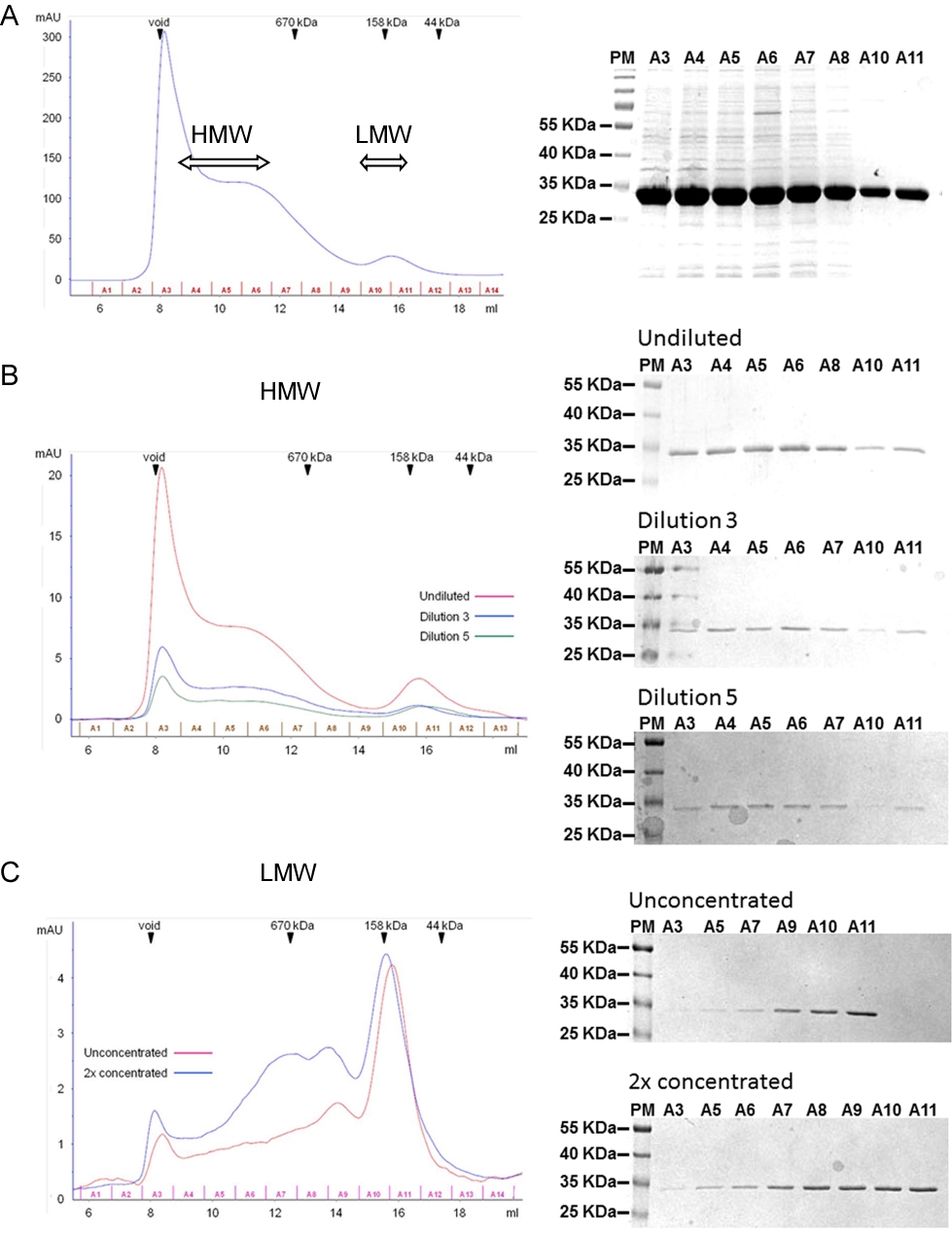

Supplement: S2 Fig — A) GF of Strep-FloAsp after Streptavidin affinity chromatography. HMW (high molecular weight) fractions A3 to A8, low molecular weight (LMW) fractions A10 and A11. Fractions in panels showing SDS-PAGE correspond to elution fractions in GF. B) GF of the HMW fraction. Different dilutions of the fraction are marked by different colours. C) GF of the LMW fraction. Because of the low yield of this fraction, it was not diluted but rather concentrated (indicated by different colour lines) to investigate concentration-dependent behavior of fraction formation. (JPG) [file pgen.1006116.s013.jpg]

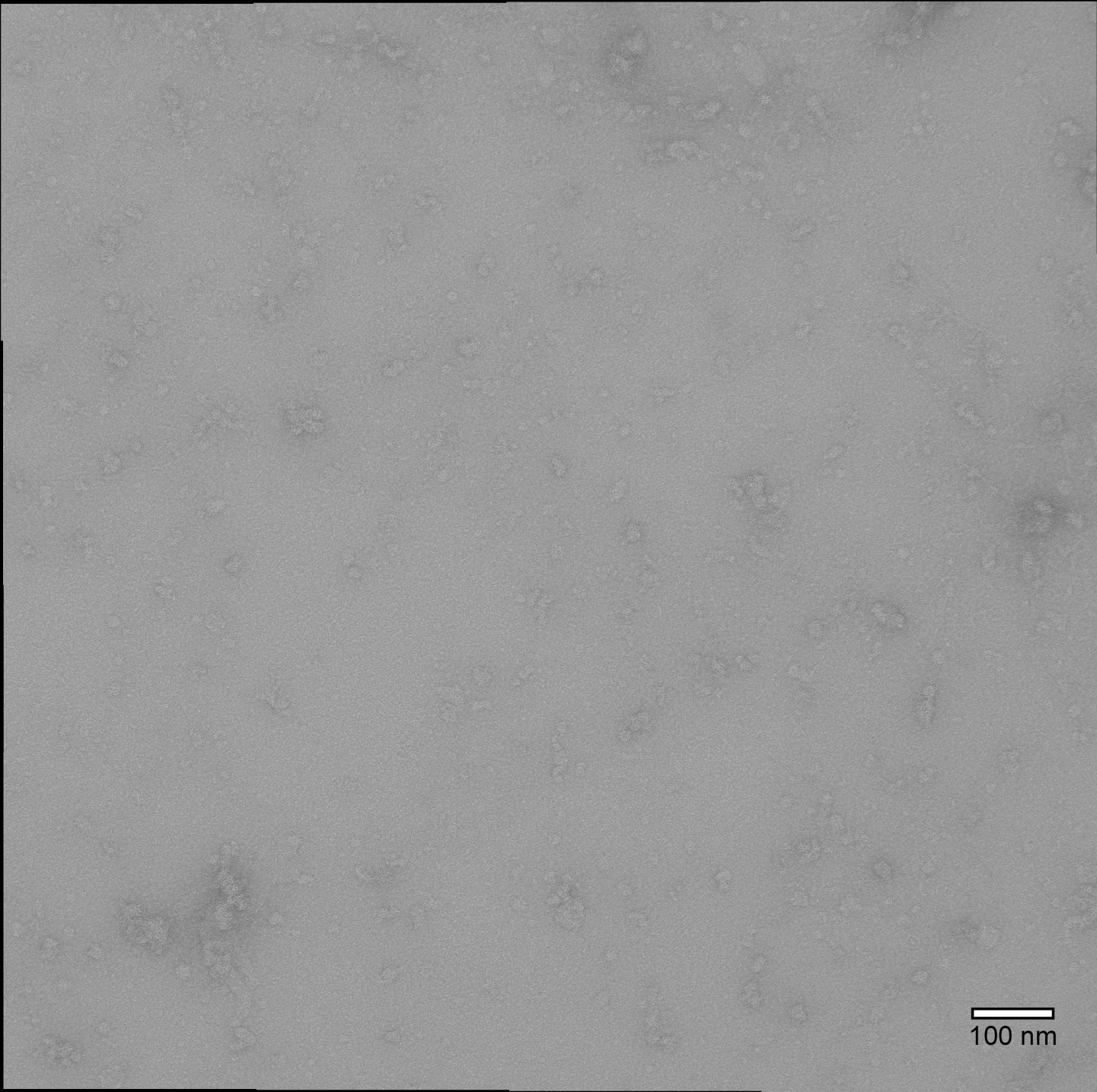

Supplement: S3 Fig — (JPG) [file pgen.1006116.s014.jpg]

## Slide 1
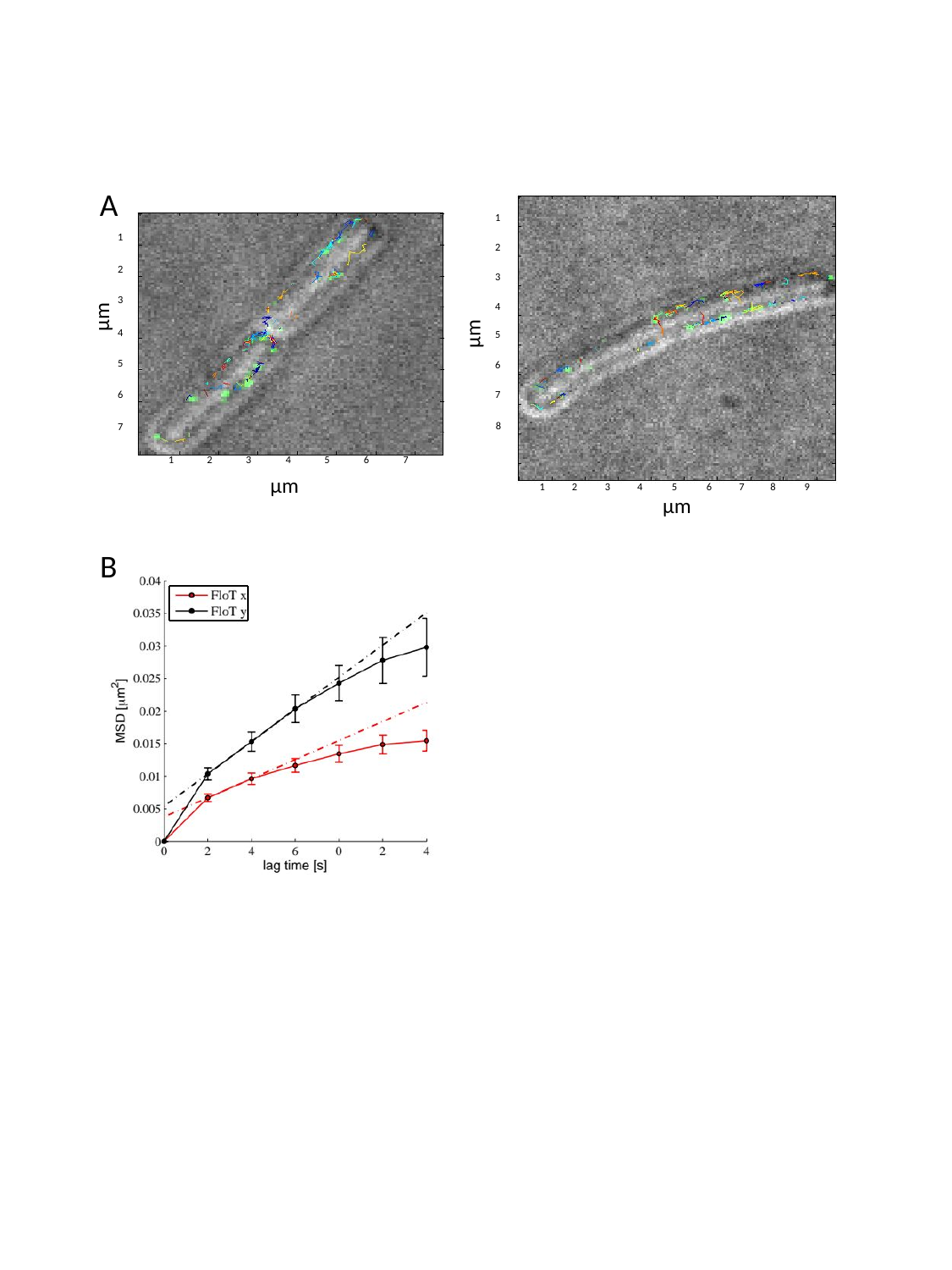

A
1
2
3
4
5
6
7
8
1
2
3
4
5
6
7
8
9
1
2
3
4
5
6
7
1
2
3
4
5
6
7
µm
µm
µm
µm
B

Supplement: S4 Fig — A) Two examples of FloA-YFP foci (green, overlaid with bright field acquisition) that are tracked over a time of 8 seconds. B) MSD plot for FloT, differentiated into x- and y-movement. (PPTX) [file pgen.1006116.s015.pptx]

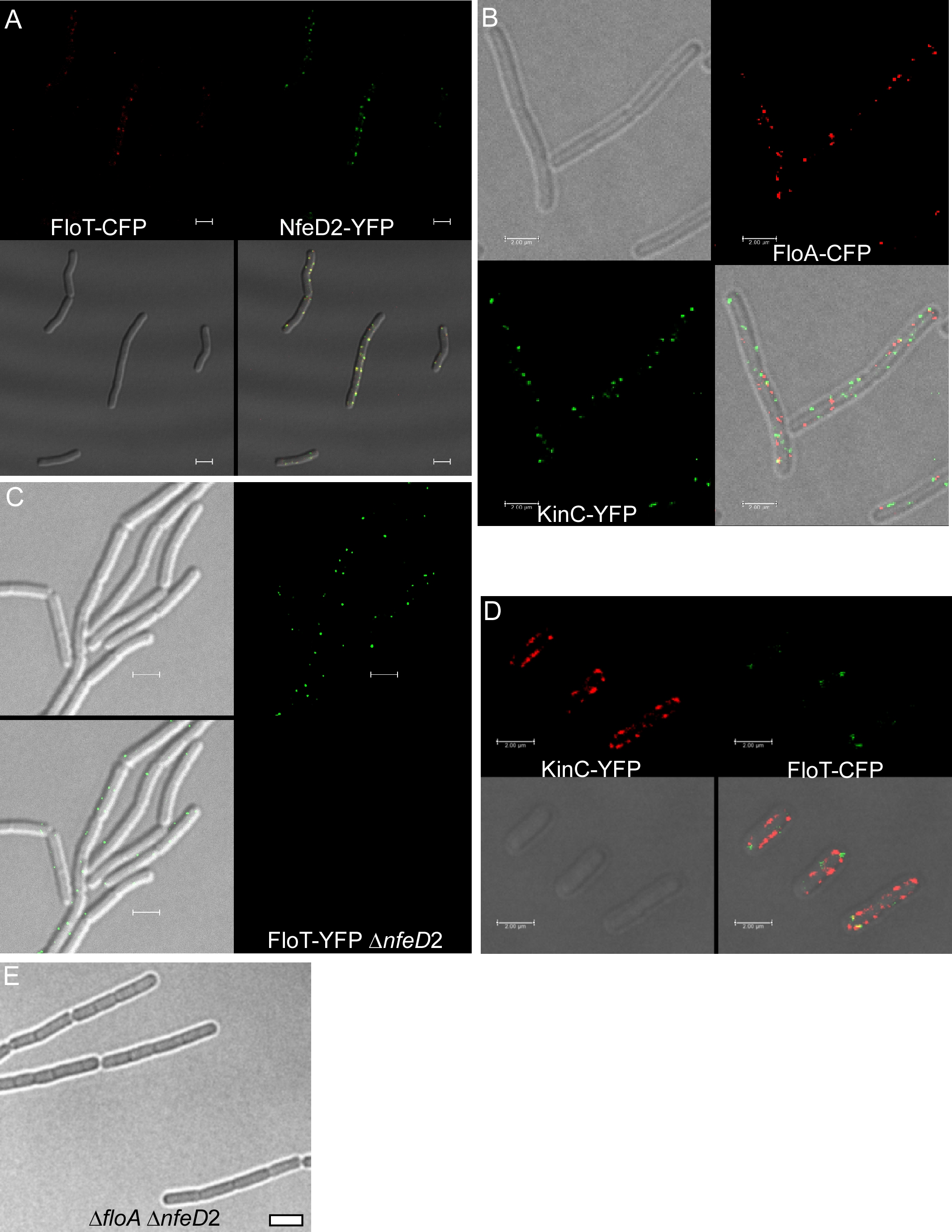

Supplement: S5 Fig — A) Colocalization of FloT-CFP and NfeD2-YFP, B) Scarce colocalization of FloA-CFP and KinC-YFP, C) localization of FloT-YFP in an nfeD2 deletion background, D) Scarce colocalization of FloT-CFP and of KinC-YFP. E) Double floA nfeD2 mutant cells. Colours of foci in overlays correspond to the single channels. Scale bars 2 μm. (JPG) [file pgen.1006116.s016.jpg]

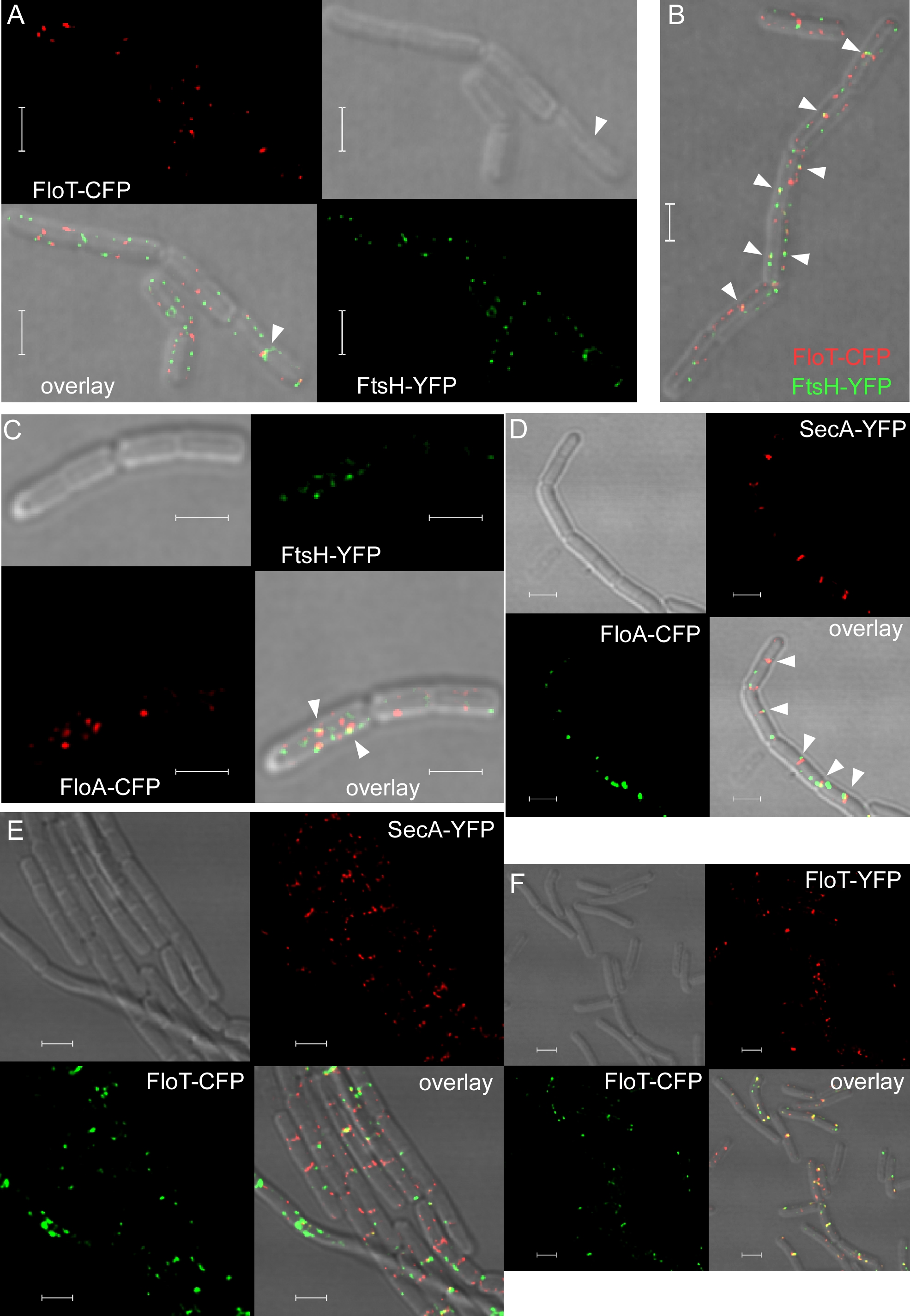

Supplement: S6 Fig — A) Confocal microscopy. FloT-CFP rarely colocalizes with FtsH-YFP. The white triangle indicates the presence of a division site, where both proteins frequently localize. B) Chain of cells with several cases of colocalization of both proteins, as seen by the yellow colour in the overlay (indicated by white triangles). Colours of foci in the overlay correspond to the colour of the labelled protein. C) Cases of colocalization of FloA-CFP and FtsH-YFP (white triangles). D) FloA-CFP colocalizes with SecA-YFP in 10 to 20% of the cases (indicated by white triangles). Colours of foci in overlays correspond to the single channels. E) FloT-CFP colocalizes with SecA-YFP in 20% of the foci. F) Colocalization of FloT-YFP expressed from the original gene locus and FloT-CFP expressed from the amylase locus. Scale bars 2 μm. (JPG) [file pgen.1006116.s017.jpg]

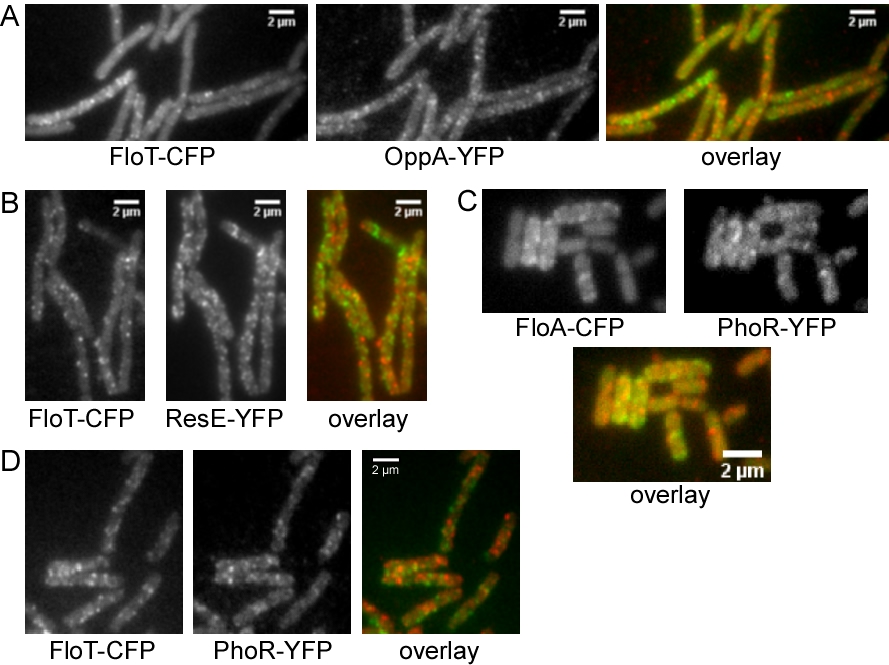

Supplement: S7 Fig — Dual colour localization studies of A) FloT-CFP together with OppA-YFP, B) FloT-CFP and ResE-YFP, C) FloA-CFP and PhoR-YFP, D) FloT-CFP and PhoR-YFP. White bars 2 μm. (JPG) [file pgen.1006116.s018.jpg]
